# Confidence limits, error bars and method comparison in molecular modeling. Part 2: comparing methods

**DOI:** 10.1007/s10822-016-9904-5

**Published:** 2016-03-04

**Authors:** A. Nicholls

**Affiliations:** OpenEye Scientific Software, Inc., Santa Fe, NM USA

**Keywords:** Statistics, Computational methods, Evaluations, Significance, Bayes, Correlation, Error bars, Confidence intervals

## Abstract

The calculation of error bars for quantities of interest in computational chemistry comes in two forms: (1) Determining the confidence of a prediction, for instance of the property of a molecule; (2) Assessing uncertainty in measuring the difference between properties, for instance between performance metrics of two or more computational approaches. While a former paper in this series concentrated on the first of these, this second paper focuses on comparison, i.e. how do we calculate differences in methods in an accurate and statistically valid manner. Described within are classical statistical approaches for comparing widely used metrics such as enrichment, area under the curve and Pearson’s product-moment coefficient, as well as generic measures. These are considered of over single and multiple sets of data and for two or more methods that evince either independent or correlated behavior. General issues concerning significance testing and confidence limits from a Bayesian perspective are discussed, along with size-of-effect aspects of evaluation.

## Introduction

Part One of this paper [1] focused on the calculation of error bars, or confidence limits for measured or calculated quantities. Such confidence intervals might have intrinsic value, e.g. is a solubility or a pKa acceptable for project X? More abstractly, a value without an indication of uncertainty lacks important information, and can be as misleading as it is informative. In the drug discovery process inaccurate data can confuse rather than guide.

Often, however, confidence limits are used to assess relative performance or merits, e.g. is a new method better or worse than an old one? Is the loss in accuracy of a faster or cheaper assay acceptable compared to a slower or more expensive approach? What is the probability a molecule’s property is actually worse than another’s, given measurement imprecision? In this case we have to assess the *accuracy of differences*, rather than just accuracy. New concepts are important here, in particular the role of covariance between methods. In Part One covariance was considered in the context of the additivity of random effects, rather than its role in differentiating performance. Procedures are presented for comparing metrics in common use in computational chemistry both when covariance, i.e. correlation, is important and when it is not.

Measuring relative performance is crucial in any field that hopes to improve its capabilities. If a field cannot assess whether a new approach is not just new but actually better how can it progress? Yet, in a competitive field, such as computational chemistry, alternate methods may derive from different, competing interests. As such it is all too easy for statistics to be either ignored or used improperly to justify ill conceived but novel methods, whether because of the need for publication, career or product advancement. As such, it is important not just to get the statistics of relative measurement correct but also to know what such statistics really mean and what they do not mean.

Two important aspects of statistics that are most commonly misunderstood are the difference between confidence limits and a test for significance, and between significance and the size of an effect. A confidence limit is a range of values that, with a given level of probability, we believe captures the actual value of a quantity. On the other hand, a significance level is the probability that observed values could have been seen if there actually was no “effect” or difference, but random chance made it appear that way. Confidence limits can be used to a similar effect, e.g. whether an estimate on the range of a difference brackets “zero”, i.e. “no difference”. Both have their utility and both can be misappropriated, misunderstood and misused. Secondly, significance does not imply importance of an effect, merely its existence. No two computational methods will have identical results. As such, the average performance of one will always be better than the other. Given enough examples, such a difference in average performance can always be found to be *significant*—but is it important? Is a difference, say, in 1 % enrichment for a virtual screen between 20.00 and 20.01 important? This distinction between significance and size-of-effect has become an important issue in many fields, e.g. economics and the social sciences, and presented here are some practical aspects of this debate for our field.

The structure of this follow-on paper is as follows:

1. Comparing two methods with known confidence limits
  - Independent errors
  - Dependent errors
  - Corrections for small sample numbers
  - Dealing with asymmetric confidence limits
  - Averaging over a set of systems
2. Binary systems
  - Probabilities
  - Virtual Screening AUCs
  - Virtual screening enrichments
3. Pearson’s r-squared
  - Independent errors
  - Dependent errors
4. Thoughts and observations on parametric vs non-parametric modeling of differences
5. Comparing multiple methods
  - Comparing a single method to a series of others
  - Determine whether a series of methods are actually equivalent to each other
  - Distinguishing single methods from a set of methods
6. Discussion of conceptual issues concerning confident intervals and significance testing

## Comparing two methods with known confidence limits

### Independent errors

**Fig. 1:**
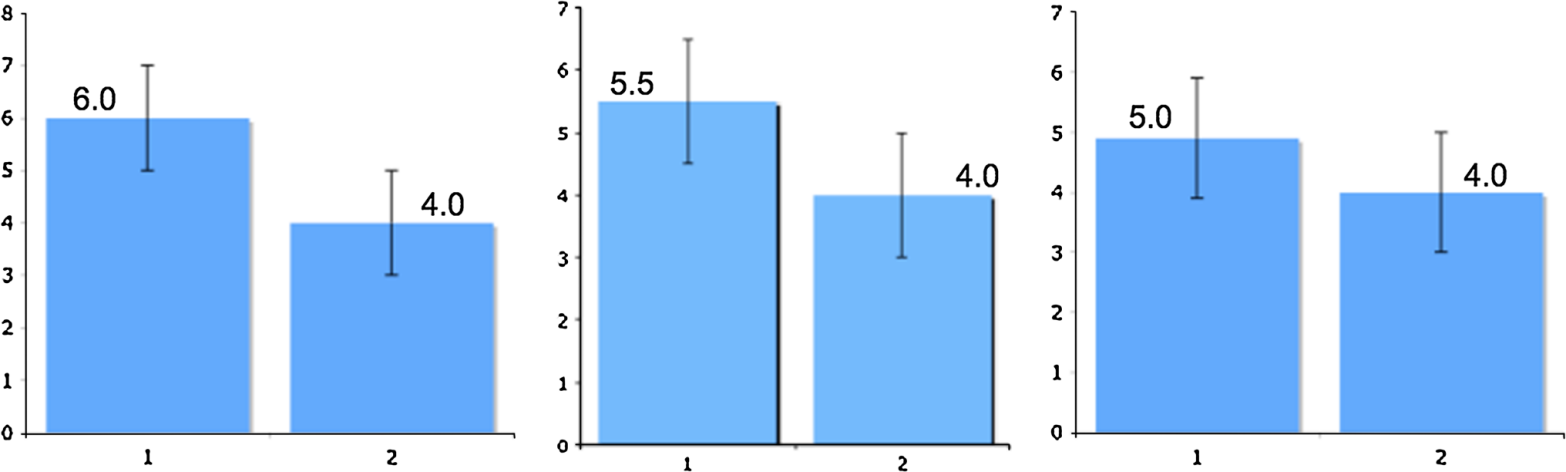
Three comparisons of histograms, each with 95 % confidence limits of 1.0. Both the left and center comparisons are statistically different at this confidence level. However, most challenged with these graphs assume only the left panel shows methods that are distinct due to the commonly quoted but incorrect “non-overlap of error bars” rule

Part One described how the variance of the difference between properties is found by summing the variance of each property. Suppose method or property A and B have equally sized error bars, then this means the error bar for the difference is simply that of A and B scaled by √2. This may seem obvious but it is contrary to a common assumption, which says that if two error bars overlap then two methods are statistically equivalent. Figure 1 shows an example of two histograms being compared, each with error bars of ±1.0. In several, admittedly uncontrolled, polls scientists were shown the triptych below and asked which panel shows methods that are statistically equivalent. A large majority mislabelled the middle panel, claiming this shows methods that are statistically similar because the error bars overlap. This would only be true if errors added in a linear fashion. As the error bar of the *difference* is 1.41 (i.e. √2) the pair in this panel are significantly different. If error bars do *not* overlap we *can* say two methods are statistically different (left panel) at the same level of the significance represented by the error bars but the converse is not correct.

### Dependent errors

The right-most panel of Fig. 1 represents method *A* with metric of performance of 5.0 and *B* of 4.0; assume larger is better. Clearly we cannot say that *A* is better than *B* with 95 % confidence because the 95 % confidence bar for the difference is 1.41 and the actual difference is only 1.0. If we calculate the probability of *A* actually being better than *B* we find that there is roughly an 80 % chance of this being so.

Now suppose this metric for *A* is an average over ten systems and that for every single system *A* performed better than *B*. If the two methods were equivalent we would expect *A* to only beat *B* five times out of ten. The odds of it beating *B* every single time are the same as the odds of flipping heads ten times out of ten, i.e. about one in a thousand. This would suggest the probability *A* is better than *B* is 99.9 %, not 80 %. So what has gone wrong here?

The cause of this discrepancy is that we have used the *same* ten systems to compare *A* and *B*. All of the statistics for the addition of errors is based on the concept that errors are independent of each other, i.e. variation in one method is independent of the variation in the other. But if the performance of *A* and *B* are related to each other they are not independent at all. The correct way to construct an error bar for a *dependent* system is on the difference between *A* and *Bper system*, i.e. the variation of the difference of A and B on each system:

1 $$Var\left( {A - B} \right) = \mathop \sum \limits_{i = 1}^{N} \frac{{\left( {(A_{i} - B_{i} ) - \left( {\overline{A - B} } \right)} \right)^{2} }}{N - 1}$$

The standard deviation of the difference between *A* and *B* is then $\surd \left( {Var\left( {A - B} \right)/N} \right)$, which should be compared to the average difference between *A* and *B*. If the variation between *A* and *B* is pretty constant, i.e. *A* moves up as does *B*, then *Var*(*A* − *B*) is small and the difference between *A* and *B* may be very significant.

We can see more clearly the consequences of the *co*-varying of A and B by reordering the last equation:

2 $$Var\left( {A - B} \right) = \frac{1}{N - 1}\mathop \sum \limits_{i = 1}^{N} \left( {(A_{i} - \bar{A}) - (B_{i} - \bar{B})} \right)^{2}$$

The variance now looks like the difference between two *vectors*, *A* and *B*, where the *i*^th^ element of either is the element *A*_*i*_ or *B*_*i*_ minus its average. As such we can write the variance as we would the difference of two vectors:

3 $$Var\left( {A - B} \right) = \frac{1}{N - 1}\left( {\left| {\vec{A}} \right|^{2} + \left| {\vec{B}} \right|^{2} - 2\left| {\vec{A}} \right|\left| {\vec{B}} \right|\cos \theta } \right)$$

Where *θ* is just the angle between the two vectors. We can rewrite this formula:

4 $$Var\left( {A - B} \right) = Var\left( A \right) + Var\left( B \right) - 2r\sqrt {Var\left( A \right)Var\left( B \right)}$$

Here *r* is actually just Pearson’s correlation coefficient. We can replace the variance by the standard deviation, σ, and obtain the formula:

5 $$var\left( {A - B} \right) = \sigma_{A}^{2} + \sigma_{B}^{2} - 2r\sigma_{A} \sigma_{B}$$

If the correlation between the two sets of results is zero (r = 0), then we get the standard result for the addition of error terms is as follows:

6 $$var\left( {A - B} \right) = \sigma_{A}^{2} + \sigma_{B}^{2} = var\left( A \right) + var\left( B \right)$$

If the variances of *A* and *B* are equal we obtain an error bar for the difference that is √2 larger than for the component methods. However, if *r* > 0, i.e. the performance of each methods are correlated, this estimate is too strict. *A* may be better than *B* even if the differences in their average performance is smaller than the independent assessment of mutual error. In theory, the variance of the difference could become zero if there is perfect correlation between results and the variances of *A* and *B* are equal. In practice, lower and upper bounds on the variance of the difference are:

7a $$var\left( {A - B} \right)_{min} = \left( {\sigma_{A} - \sigma_{B} } \right)^{2}$$

7b $$var\left( {A - B} \right)_{max} = \left( {\sigma_{A} + \sigma_{B} } \right)^{2}$$

**Fig. 2:**
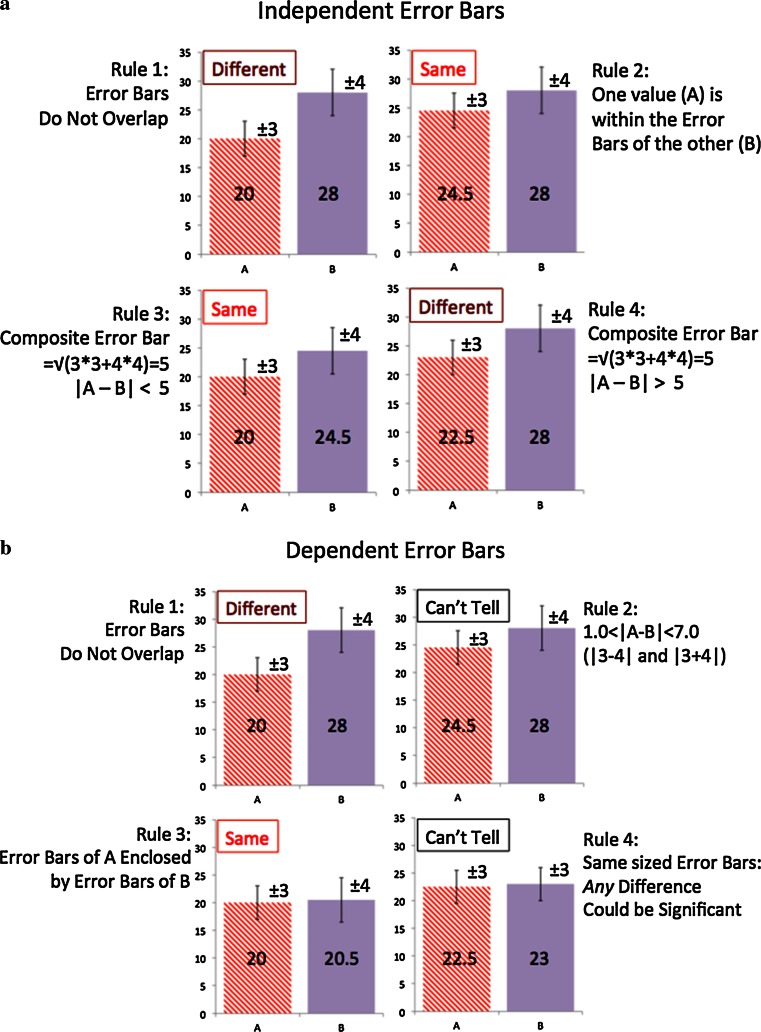
These figures illustrate the rules for statistical deduction from standard *bar charts* with *error bars*. The *error bars* are for 95 % significance and are ±3.0 for Method A and ±4.0 for method B, giving a composite independent *error bar* of ±5.0. The single exception is in the *bottom right* figure for dependent *error bars* where both are set to ±3.0

This means the maximum and minimum of the difference error bars is simply the sum and difference of the individual error bars respectively. This illuminates the real rules for error bars. If the error bars of two measures do not overlap they are concretely different, no matter the correlation. Similarly, if the error bars of one measure lie within the error bars of the other measure then the two methods cannot be statistically different, even if they are maximally correlated. These rules are described succinctly below and illustrated in Fig. 2a, b. *Independent* measures (e.g. tested against different datasets):

$$Composite\;Error = \sqrt {\left( {Size\;of\;Error\;Bar1} \right)^{2} + \left( {Size\;of\;Error\;Bar2} \right)^{2} }$$

1. If the error bars do not overlap the measures are statistically different (because the composite error must be less than the sum of the two error bar sizes)
2. If one measure lies within the error bar of the other then the measures are not statistically different (the composite error is always larger than either error bar)
3. Two measures are not significantly different if they are closer than their composite error bar
4. Two measures are significantly different if they are further apart than their composite error bar

*Dependent* measures (e.g. tested against the same datasets):

$$Composite\;Error < Size\;of\;Error\;Bar1 + Size\;of\;Error\;Bar2$$

$$Composite\;Error > \left| {Size\;of\;Error\;Bar1 - Size\;of\;Error\;Bar2} \right|$$

1. If the error bars do not overlap the measures are statistically different
2. To be distinguishable measures must differ by more than the sum or absolute difference of the sizes of the individual error bars.
3. If the error bars of one measure lie entirely within the error bars of the other then these two measures cannot be statistically different, no matter what their correlation.
4. If the error bars are the same size then *any* difference in mean values could be statistically significant

Anti-correlated measures are always nice to find, e.g. if we know that method *A* will succeed when method *B* fails and vice versa. Averages of such methods are then more robust. However, correlated measures are more common than anti-correlated measures, e.g. we are often comparing small variants of methods. As such, it can be very helpful to include method-to-method correlation if you wish to statistically prove progress in the development of a procedure. In the statistical literature this approach to accounting for the correlation between two methods is often referred to as the *paired Student ttest* [2]. This is described more in the next section regarding small sample sizes.

### Corrections for small sample numbers

The above description of error combination assumes we are in the asymptotic limit of large numbers of samples. Often we have small numbers of samples. Suppose, for instance, we want to compare the property difference for two compounds, *A* and *B*, where that property has been measured in triplicate, i.e. the sample size is just three. Suppose we ignore any knowledge of the expected variance of the experiment, i.e. we have to estimate the standard deviation from the three measurements; from Part One we know that for *N* = 3 we are required to use the Student *t*-distribution. In this case the correct *t* statistic would be:

8 $$t = \frac{{X_{A} - X_{B} }}{{\sqrt {\left( {var\left( A \right) + var\left( B \right)} \right)/N} }} = \frac{{X_{A} - X_{B} }}{{\sqrt {\left( {var\left( A \right) + var\left( B \right)} \right)/3} }}$$

where:

9 $$var\left( {\alpha = A\;or\;B} \right) = \frac{1}{N - 1}\mathop \sum \limits_{i = 1}^{i = 3} \left( {x_{i}^{\alpha } - \overline{{x_{\alpha } }} } \right)^{2} = \frac{1}{2}\mathop \sum \limits_{i = 1}^{i = 3} \left( {x_{i}^{\alpha } - \overline{{x_{\alpha } }} } \right)^{2}$$

I.e. using the “*N* − 1” unbiased estimator of variance. The question arises, however, as to the number of degrees of freedom, *ν*, which is necessary in order to set the *t* threshold for significance. Is it *ν* = 2? This doesn’t seem right because there are six measurements. In fact, the correct answer is *ν* = 4, because there are six measurements but two estimations of means (for *A* and *B*) being used. This makes a significant difference since *t*_95%_(*ν* = 2) = 4.30 and *t*_95 %_(*ν* = 4) = 2.78.

If there are unequal numbers of measurements for *A* and *B*, namely *N*_*A*_ and *N*_*B*_, the formula for t is slightly different:

10 $$t = \frac{{X_{A} - X_{B} }}{{\sqrt {\frac{1}{{N_{A} }} + \frac{1}{{N_{B} }}} \sqrt {\frac{{\left( {N_{A} - 1} \right)var\left( A \right) + \left( {N_{B} - 1} \right)var\left( B \right)}}{{N_{A} + N_{B} - 2}}} }}$$

This looks different to Eq. 8 but reduces to the same expression when *N*_*A*_ = *N*_*B*_. The number of degrees of freedom used to calculate t_95 %_ is *ν* = *N*_*A*_ + *N*_*B*_ − 2.

Finally, the above description assumes that the standard deviations for both sets of measurements is supposed to be the same, i.e. what we have done is to combine the estimates of variance from the two sets of measurements to improve the accuracy of our estimate. If we know, or if we wish to assume, this is not true (that the methods have different variances) then the formula for *t* is:

11 $$t = \frac{{X_{A} - X_{B} }}{{\sqrt {\frac{var\left( A \right)}{{N_{A} }} + \frac{var\left( B \right)}{{N_{B} }}} }}$$

This is the standard combination of variance we would expect from the combination of independent errors. The calculation of the degrees of freedom, however, is more complicated and requires the *Welch*–*Satterthwaite* formula [3] described in Part One.

As a final note, a word of caution needs to be given to the estimation of the variance from small samples. In the case above we considered the example of having only three observations from which to deduce a variance for a measurement. The formula for the standard deviation that we use is the best estimate of the variance *assuming no prior knowledge*. However, it is unlikely this is the case. For instance, if we know the instrument or procedure used to measure the properties is accurate then we may be able to use that known error. If we have considerable prior knowledge as to the likely variance of the measurements we are free to use it. Bayesian techniques give a natural framework for including known estimations of variance with current observations but such approaches are beyond the scope of this article.

### Asymmetric error bars

In Part One it was discussed as to how confidence limits might not be symmetric, though we often treat them as if they were. An example would be if there is a natural bound on a property, e.g. zero for a root-mean-square-error, or zero and one for a probability. We will later consider the case of Pearson’s correlation coefficient in detail later, but the general prescription is as follows:

1. If there is a transformation that renders the likely distribution of values as Gaussian, then calculate the error bars in this transformed space.
2. Apply the same rules as above to the transformed error bars, i.e. two methods are different, the same or not distinguishable when their difference (in transformed values) are compared to the composite error bar (in transformed coordinates).
3. If necessary, transform the symmetric error bars back to the original function space and apply these (asymmetric) error bars to the difference of original values.

For independent errors there are also some convenient approximations. Suppose the range of *A* is: [*X*_*A*_ − *L*_*A*_, *X*_*A*_ + *U*_*A*_] and that of *B* is: [*X*_*B*_ − *L*_*B*_, *X*_*B*_ + *U*_*B*_], where *X*_*A*_ > *X*_*B*_, then [4]:

12 $$X_{A} - X_{B} \in \left[ {X_{A} - X_{B} - L, X_{A} - X_{B} + U} \right]$$

where:

13 $$L = \sqrt {L_{A}^{2} + U_{B}^{2} }$$

14 $$U = \sqrt {L_{B}^{2} + U_{A}^{2} }$$

Essentially these equations arise by considering the two sides of the error bars to be from different Gaussians. The lower “Gaussian” of A combines with the upper “Gaussian” of B to describe the case where the difference between A and B is less than average, and the reverse combination describes when the separation between A and B is bigger than expected. One then compares (*X*_*A*_ − *X*_*B*_) to the lower bound, *L*, to test significance against the null model.

### Averaging over a set of systems

Typically we measure the performance of some method over a set of systems for which both the performance and variability are not constant. In Part One we considered how the system-to-system variability should be combined to arrive at confidence limits for a method over all systems. These confidence limits can be treated as above when comparing methods. In addition, Part One described how, for instance, if we were looking at how variable a docking method is over a set of proteins we can adjust (lower) this variability by accounting for how imprecise we think our estimate of performance is on each particular protein. I.e. noise in each system, for instance due to a finite number of active compounds, contributes to the variability over *all* systems.

However, if we are comparing two methods over the same set systems we need to determine the *correlated* variance for *each* system. Then, we look at the variability of the *difference* in performance over a set of systems with which to adjust the variability of the whole set. As a concrete example, suppose we are docking to a set of proteins by methods A and B and are calculating the Area-Under-the-Curve (AUC) as a metric of performance. It is not enough to calculate the protein-by-protein error in the AUC for A and B separately; we need to know the effects of correlation on the difference in AUC for each protein.

Both AUC and enrichment are examples of averages over binary events; in the case of the AUC it is whether a randomly chosen “active”, e.g. a compound that passes some experimental threshold, scores higher than a randomly chosen inactive, e.g. a compound that fails to pass this threshold. In the case of enrichment, it is whether an active falls within a certain range of compound ranking by some algorithm. Both represent “binary” events. Calculating the effects of correlation between two such measures is considered in the next Section. 

## Binary systems

### Probabilities

From Part One we know the formula for the variance of a single probability *p* is very simple, i.e. it is merely *p*(1 − *p*). But what is the variance of the difference of two probabilities? Suppose that we have calculated *p* by averaging a binary variable, i.e. either 1 or 0, over *N* examples. We could form a *difference* variable by subtracting the binary variable for B from the binary variable for A. Two ones give zero, two zeros give zero and a one and a zero gives plus or minus one, depending on the order. This gives us a new variable that is no longer binary; it can be one, zero or negative one. We can calculate a variance for this new variable by the usual methods. In particular, we can use the formula from above:

15 $$var\left( {A - B} \right) = var\left( A \right) + var\left( B \right) - 2r\sqrt {var\left( A \right)var\left( B \right)}$$

where now:

16 $$var\left( A \right) = p_{a} \left( {1 - p_{a} } \right)$$

17 $$var\left( B \right) = p_{b} \left( {1 - p_{b} } \right)$$

And *r* is the Pearson’s correlation coefficient between the two binary vectors. I.e. there is no conceptual difficulty in calculating the correlated variance between two probabilities, which may be greater or less than the uncorrelated quantity depending on whether the instances that make up the probability calculation are positively or negatively correlated. This is the approach we take to consider virtual screening metrics.

There is a special case that can be useful. Suppose we want to compare probabilities for two classifications, e.g. *p*_*a*_ is the probability of category *A* and *p*_*b*_ is the probability of category *B* and A and B are the only categories, for instance perhaps *A* everything that passes a threshold, and *B* is everything that does not. The probability vectors described above for *A* and *B* are anti-correlated, i.e. whenever *A* is a 1, *B* is a 0 and vice versa. This means that *r* = −1.0. Furthermore, since *p*_*a*_ + *p*_*b*_ = 1, *var*(*A*) = *Var*(*B*) in the above example. This leads to the very simple formula for the net variance:

18 $$var\left( {A - B} \right) = 4 \times var\left( A \right)$$

This leads to a very simple test as to whether a given yes–no distribution is statistically different from 50:50. If *p*_*a*_ = *p*_*b*_ = 1*/*2 then:

19 $$var\left( {A - B} \right) = 4 \times \frac{1}{2} \times \left( {1 - \frac{1}{2}} \right) = 1$$

Therefore, if there are *N* samples, we expect 95 % of the variation from 50:50 to be ±1.96/√*N* ≈ ±2/√*N*. If we have *ΔN* as the difference in *yes*–*no*, *up*–*down* votes for this binary example then for this difference to be significant at the 95 % level we require:

20 $$\begin{aligned} \left( {\Delta N/N} \right) > 2/\sqrt N \hfill \\ \left( {\Delta N} \right)^{2} > 4N \hfill \\ \end{aligned}$$

For instance, if 120 active compounds are examined and 70 have a feature *X* and 50 do not. Is it statistically significant that active compounds have *X*? Here, *N*_*A*_ = 70 and *N*_*B*_ = 50, the square of the difference is 400. The right hand side is 4 × 120 = 480; therefore this difference is not significant at the 95 % level.

### AUC

This concept of the different in binary vectors is straightforward to apply to the calculation of a correlated AUC. The components that go into the variance of an AUC are the variance of the probability for an active scoring higher than an inactive, plus the variance of the probability an inactive scores higher than an active [5, 6]. When we are looking at the correlated difference between two AUCs we merely have to look at how to calculate the correlated difference of these components.

This can be done by considering the contribution to the total variance of each actives/inactives in turn, using the above approach to calculate the covariance. I.e. suppose we select a single active compound and make a vector of length *N*_*I*_, where *N*_*I*_ is the number of inactives. Then we go down the list of inactives and place a “1” in the vector every time the active scores higher than this inactive using method *A* and “0” otherwise. This is our binary vector for this active corresponding the example above. The average of all entries in the vector is the probability, *p*, that this active scores higher than a randomly chosen inactive. The average variance is therefore *p*(1 − *p*).

The average of all such variances is the first term in the expression from Delong et al. for the variance of the AUC [6]. However, we can repeat this procedure to generate a second vector for this active by using Method *B*. With these two vectors we can form a product vector that represents the correlation of the two methods for this active, i.e. the angle between the two binary vectors representing whether inactives score higher or lower using method *A* or method *B*. This can be repeated over all actives, and also over all inactives, where the vector is then of length equal to the number of actives and the binary condition is whether the inactive scores higher then an active. If both methods tend to rank the same active or inactive in similar ways then it will reduce the total variance in the DeLong formula, or increase it if they rank in very different ways.

Formally, then, the correlated version of the DeLong formula looks like this:

21 $$\begin{aligned} Err_{AUC, A - B}^{2} = & \frac{{var_{active}^{A} + var_{active}^{B} - 2cov_{active}^{AB} }}{{N_{active} }} \\ & \quad + \frac{{var_{inactive}^{A} + var_{inactive}^{B} - 2cov_{inactive}^{AB} }}{{N_{inactive} }} \\ \end{aligned}$$

where:

22 $$cov_{active} = \frac{1}{{N_{Actives} - 1}}\mathop \sum \limits_{i = 1}^{{N_{Actives} }} \left[ {\frac{1}{{N_{Inactives} }}\left( {\mathop \sum \limits_{j = 1}^{{N_{Inactives} }} \chi_{i,j}^{A} \chi_{i,j}^{B} } \right) - p_{i}^{A} p_{i}^{B} } \right]$$

Here, *χ*_*i*,*j*_ is one if active *i* scores higher than inactive *j*, otherwise zero and *p*_*i*_ is the average of this quantity, i.e. the probability active *i* scores higher than any inactive. The formula for the covariance of the inactives follows the same form with the appropriate quantities swapped.

### Enrichment

The same logic can be applied to the calculation of the composite error of two ROC enrichments, i.e. where the enrichment is defined as relative to the fraction of inactives, not the fraction of total compounds [7]. If the same (actives/inactives) are discovered in the top *X* % of the list then two methods are highly correlated and the error bars for their difference in performance for that system need to reflect this fact. The binary vector upon which we perform our operations is simpler than in the case of the AUC. All we have to consider is whether an active or an inactive falls above the enrichment threshold and form two binary vectors, one for actives and one for inactives. From Part One variance of each contributes to the total variance of the enrichment:

23 $$var\left( {e_{A\;or\;B} \cdot f} \right) = \frac{{g_{A\;or\;B} \left( {1 - g_{A\;or\;B} } \right)}}{{N_{Actives} }} + S_{A\;or\;B}^{2} \frac{{f\left( {1 - f} \right)}}{{N_{Inactives} }}$$

Here *S* is the slope of the ROC curve at *f*, the fraction of inactives found, and *g* is the fraction of actives and the variance is calculated for the enrichment scaled by *f* so as to have the form of a probability (i.e. from 0 to 1). From this the formula for the square of the error of the difference of two enrichments has the form:

24 $$\begin{aligned} Err_{Enrichment, A - B}^{2} = & \frac{1}{{fraction\_inactives^{2} }}\left( {\frac{{var_{active}^{A} + var_{active}^{B} - 2cov_{actives} }}{{N_{active} }}} \right. \\& \left. {\quad + \frac{{S_{A}^{2} var_{inactive}^{A} + S_{B}^{2} var_{inactive}^{B} - 2S_{A} S_{B} cov_{inactives} }}{{N_{inactive} }}} \right) \\ \end{aligned}$$

where,

25 $$cov_{Actives} = \frac{1}{{N_{active} }}\left( {\mathop \sum \limits_{i = 1}^{{N_{active} }} \chi_{i}^{A} \chi_{i}^{B} } \right) - g_{A} g_{B}$$

Here, *χ*_*i*_ is 1 if active *i* is in the top fraction, otherwise it is zero. The formula for the covariance of the inactives follows the same form with the appropriate replacement of quantities.

**Fig. 3:**
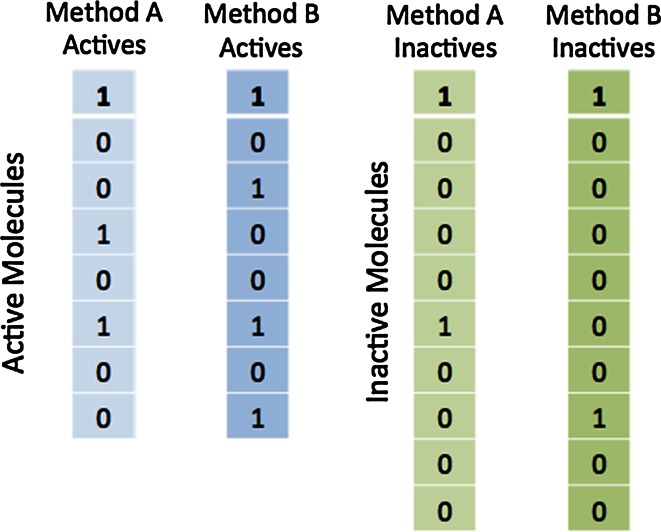
Binary vectors constructed for two methods, *A* and *B* over a set of eight actives and ten inactives. Bits are set for the inactive compounds if they are the highest or second highest ranked inactive compounds and for the active compounds if they score higher than the second ranked inactive

We illustrate this procedure of considering binary vectors representing active and inactive molecules in Fig. 3. Here we are looking at ROC enrichment at 20 %, i.e. for actives that score higher than the top two inactives.

We have:

26 $$p_{active}^{A} = \frac{3}{8};\quad p_{active}^{B} = \frac{4}{8}$$

Thus, ROC enrichments are:

27 $$\begin{aligned} E_{20\% }^{A} = & \frac{{\frac{3}{8}}}{{\frac{1}{5}}} = \frac{15}{8} = 1.875 \\ E_{20\% }^{B} = & \frac{{\frac{4}{8}}}{{\frac{1}{5}}} = \frac{20}{8} = 2.5 \\ \end{aligned}$$

The variances are:

28 $$\begin{aligned} var_{active}^{A} = & \left( {\frac{3}{8} \times \frac{5}{8}} \right) = 0.234; \quad var_{active}^{B} = \left( {\frac{4}{8} \times \frac{4}{8}} \right) = 0.25 \\ var_{inactive}^{A} = & \left( {\frac{2}{10} \times \frac{8}{10}} \right) = 0.16;\quad var_{inactive}^{B} = \left( {\frac{2}{10} \times \frac{8}{10}} \right) = 0.16 \\ \end{aligned}$$

Finally, the covariances are:

29 $$\begin{aligned} cov_{active}^{AB} = & \left( {\frac{2}{8}} \right)\sqrt {var_{active}^{A} var_{active}^{B} } = \frac{2}{64 \times 8} \sqrt {15 \times 16} = 0.061 \\ cov_{inactive}^{AB} = & \left( {\frac{1}{10}} \right)\sqrt {var_{inactive}^{A} var_{inactive}^{B} } = \frac{1}{100 \times 10} \sqrt {16 \times 16} = 0.016 \\ \end{aligned}$$

For such a simple example the slopes of the ROC curve may not be very accurate but Eq. 30 can approximate them:

30 $$\begin{aligned} S_{A} = & \frac{3/8}{2/10}\left( {1 + \frac{{\ln \left( {\frac{15}{8}} \right)}}{{\ln \left( {\frac{2}{10}} \right)}}} \right) = 1.14 \\ S_{B} = & \frac{4/8}{2/10}\left( {1 + \frac{{\ln \left( {\frac{20}{8}} \right)}}{{\ln \left( {\frac{2}{10}} \right)}}} \right) = 1.07 \\ \end{aligned}$$

Putting this all together we arrive at:

31 $$\begin{aligned} Err_{Enrichment, A - B}^{2} =& 1.96 \hfill \\\Delta Enrichment\left( {A,B} \right) =& 0.625 \pm 1.4 \hfill \\ \end{aligned}$$

Thus, the difference in enrichment in this toy problem is not significant, which is not unexpected given the small sample sizes.

## Pearson’s r-squared

It would seem particularly important to consider the comparison of Pearson’s *r* or *r*^2^ values because in computational chemistry this is typically how claims are made for method superiority. As before, cases have to be made for independent values, e.g. ones made on different test cases, as well as the much more usual situation where different methods are being applied to the same dataset, in which case *r* values may be correlated with each other, i.e. we have to consider the “correlation of correlation”. There have been many papers on this subject, going back to early work by Pearson himself [8]. Much of this work has been developed and applied in the social sciences [9–11]. Here, illustrations will be made with simulations of a simple system where two variables, *y* and *z* are correlated to a primary variable, *x*.

**Fig. 4:**
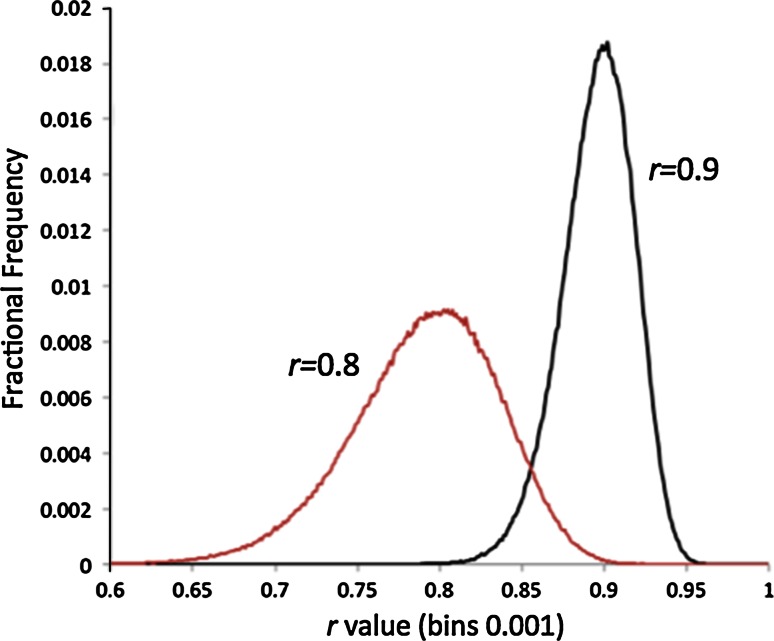
Distributions of *r* values with modal averages of 0.8 and 0.9. *Graphs* were produced by random sampling of the correlation coefficient of 50 points generated by Eq. 32, where the *x* coefficient was evenly spaced between 0.0 and 4.0. *Graphs* are made from 10^6^ independent simulations of Eq. 32, with *γ* = 0.595 for *r* peaking at 0.9 and *γ* = 0.925 for *r* peaking at 0.8

The results of the first two such simulations are shown in Fig. 4 (Fig. 8 in Part One). This is a frequency plot of 10^6^*r*_*xy*_ values from the correlation of fifty evenly spaced values from *x* = 0 to *x* = 4.0, each with a Gaussian noise added to produce fifty *y* values, i.e.:

32 $$y_{i} = x_{i} + \gamma {\mathcal{N}}\left( {0,1} \right)_{y,i}$$

The noise component, *γ*, has been scaled to produce distributions peaked at *r* = 0.8 (γ = 0.925) and *r* = 0.9 (γ = 0. 595).

Here *x* is acting as some experimental property to be predicted, *y* is some theoretical prediction that correlates with *x* except it is inaccurate, i.e. has noise of strength *γ*. Nothing would change in the results below if the constant of proportionality (e.g. slope between *x* and *y*) where not one, or if there were an offset to *y*, because the definition of a correlation coefficient is independent of such.

For our purposes we want to derive the error bounds on a difference between two *r* values. The first aspect to note is that the distributions shown in Fig. 4 are quite asymmetric. As described in section above, this does not in and of itself represent a problem. Equations 12 to 14 describe how to combine the upper and lower confidence limits to arrive at a consensus upper and lower bounds for independent measures. We can calculate these for any *r* values using the standard techniques described in Part One, e.g. using the Fisher *z*-transform which makes the distributions Gaussian, find confidence limits in *z*-space and then transform them back to *r* values [12]. For the examples shown in Fig. 4 the upper and lower 95 % confidence limits are:

33 $$U_{0.8} = 0.882; \quad L_{0.8} = 0.671; \quad U_{0.9} = 0.942; \quad L_{0.9} = 0.829$$

Putting these into Eqs. 13 and 14 for the upper and lower bounds on the distribution of the difference between *r* = 0.9 and *r* = 0.8, i.e. <*Δr*> = 0.1 gives:

34 $$\begin{aligned}&\Delta r \in \left[ {0.1 - 0.1085, 0.1 + 0.1356} \right] \hfill \\&\Delta r \in \left[ { - 0.0085, 0.2356} \right] \hfill \\ \end{aligned}$$

As *Δr* = 0.0 lies (just) within this range we can not exclude, at 95 % confidence, that the two *r* values are actually different.

Of course, we calculated the upper and lower bounds by using the Fisher trick of transforming the variables to give distributions that are closer to Gaussian in character. As such, another perfectly acceptable way to determine if the two *r* values are different would be to calculate if the values in *z*-space are actually different. If the methods are independent we know that the variance in this transformed space will be simply twice the component variances, i.e. twice 1/(*N* − 3). We then just have to compare this value to the difference between the *transformedr* values. In the case considered above we have:

35 $$\begin{aligned} &r = 0.8 \to F\left( r \right) = 0.5\ln \left( {1 + 0.8/1 - 0.8} \right) = 1.099 \hfill \\& r = 0.9 \to F\left( r \right) = 0.5\ln \left( {1 + 0.9/1 - 0.9} \right) = 1.472 \hfill \\& F\left( {0.9} \right) - F\left( {0.8} \right) = 0.373 \hfill \\ \end{aligned}$$

Meanwhile, the combined variance in the transformed space is 2/(*N* − 3),

36 $$\begin{aligned}& var\left( {F\left( {0.9} \right) - F\left( {0.8} \right)} \right) = \frac{1}{N - 3} + \frac{1}{N - 3} = \frac{2}{47} = 0.0425 \hfill \\& \frac{{F\left( {0.9} \right) - F\left( {0.8} \right)}}{{\sqrt {var} }} = \frac{0.373}{0.206} = 1.81 < t_{95\% } \left( { = 2.01\;for\;N = 50} \right) \hfill \\ \end{aligned}$$

Therefore the difference in *r* values is not quite statistically significant at the 95 % level, as we surmised from the combining of the asymmetric error bars for *r*. Note that this method only gives us the likely significance of one *r* being different than the other, not what the error bounds are on the difference.

The key assumption in the above result is that the *r* values are independent. In fact, this is rarely the case, especially when the correlations are with respect to the same underlying variable, in this case ‘*x*’. This is likely the most common use-case, e.g. two methods try to predict a single property, *x*. In this case, can two *r* values actually be independent? I.e. if two measures correlated with a third, do they not have to correlate with each other?

In fact, there is a fundamental reason such correlation coefficients cannot be independent: (1 − *r*) is a *metric* distance. In the mathematical sense this means it obeys the triangle equality:

37 $$\left( {1 - r_{xy} } \right) + \left( {1 - r_{xz} } \right) > \left( {1 - r_{yz} } \right) > \left| {\left( {1 - r_{xy} } \right) - \left( {1 - r_{xz} } \right)} \right|$$

Which simplifies to:

38 $$1 - \left| {r_{xy} - r_{xz} } \right| > r_{yz} > r_{xy} + r_{xz} - 1$$

In the case above of *r* = 0.8 and *r* = 0.9, this means:

39 $$0.9 > r_{yz} > 0.7$$

Can we go further than this and, in fact, estimate *r*_*yz*_ from just *r*_*xz*_ and *r*_*xy*_? Simple arguments show that we can and that if the underlying noise in *y* and *z*, as regards to predicting *x* are independent then:

40 $$\langle r_{yz} \rangle \approx r_{xy} r_{xz}$$

**Fig. 5:**
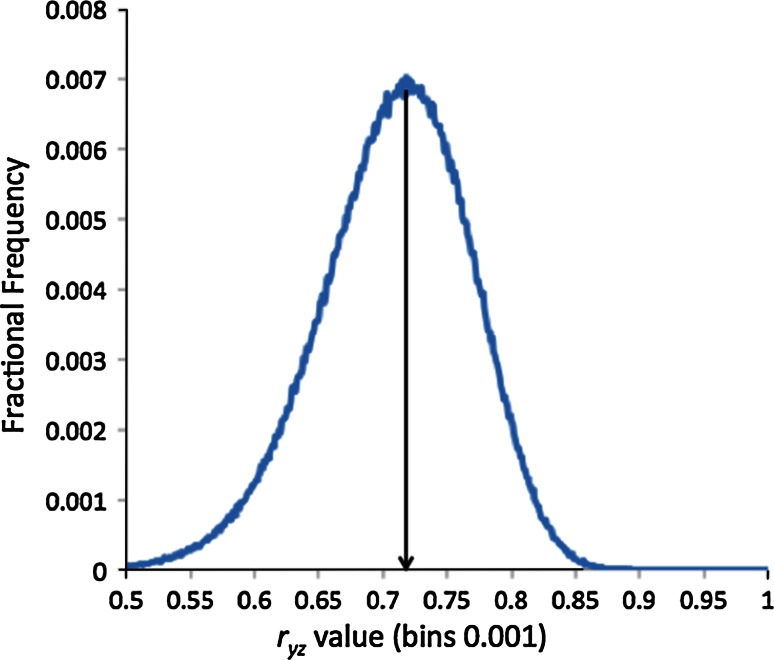
The distribution of the inter-method correlation coefficient *r*_*yz*_ for two methods *y* and *z* with correlations of *r* = 0.9 and *r* = 0.8 respectively with *x* and independent noise terms. Note the *peak* falls exactly at 0.72, the product of *r*_*xy*_ (0.9) and *r*_*xz*_ (0.8)

Shown in Fig. 5 is the frequency of method–method correlations derived from two methods described by:

41 $$\begin{aligned} y_{i} = x_{i} + 0.595{\mathcal{N}}\left( {0,1} \right)_{y,i} \hfill \\ z_{i} = x_{i} + 0.925{\mathcal{N}}\left( {0,1} \right)_{z,i} \hfill \\ \end{aligned}$$

The Pearson coefficients are 0.9 and 0.8, respectively, and the mean inter-method correlation is, as predicted, *r* = 0.72.

This gives us a yardstick as to whether two methods are merely correlated because they both correlate to the same variable, in this case *x*, or because there is some ‘deeper’ similarity. For instance, suppose *y* and *z* had the following forms:

42 $$\begin{aligned} y_{i} = & x_{i} + \gamma {\mathcal{N}}\left( {0,1} \right)_{y,i} + \eta {\mathcal{N}}\left( {0,1} \right)_{z,i} \\ z_{i} = & x_{i} + \delta {\mathcal{N}}\left( {0,1} \right)_{{z,i}} + \varepsilon {\mathcal{N}}\left( {0,1} \right)_{{y,i}} \\ \end{aligned}$$

Here each method shares a fraction of each other’s error term. Clearly the methods will be more correlated than we would expect from Eq. 41 because any resampling of the variables *y* and *z* will tend to move in the same direction; we would expect *r*_*xy*_ and *r*_*xz*_ to adjust in the same direction.

**Fig. 6:**
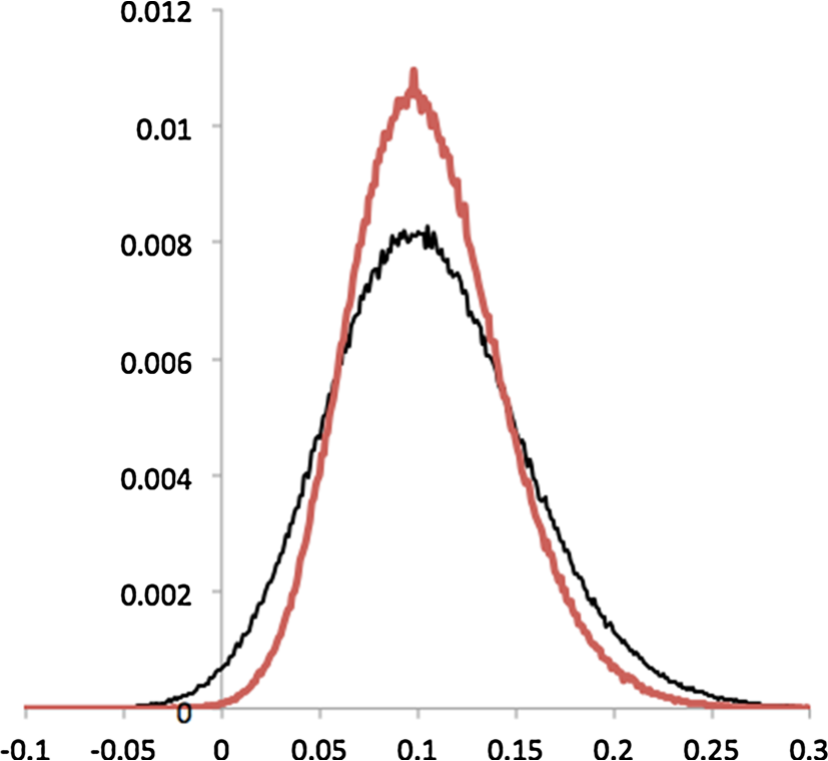
Distribution of the difference in *r* values. In *black* (*wider*) is the distribution of differences between two correlation coefficients with independent error terms. In *red* (*narrower*) is the difference in correlation coefficients that have dependent error terms. Note that the distribution of the difference of *r* values is reasonably symmetric. In both cases the mean values of the underlying correlation coefficients is 0.8 and 0.9

Figure 6 shows the distribution of the differences between *r*_*xy*_ and *r*_*xz*_ for the parameter sets:

1. *y*(*γ*, *η*) = (0.595,0) and *z*(*δ*, *ε*) = (0.925,0)
2. *y*(*γ*, *η*) = (0.595,0) and *z*(*δ*, *ε*) = (0.595,0.708)

Both sets of parameters were tuned to produce an average *r* of 0.9 for *y* and 0.8 for *z* (i.e. for the second set, 0.595^2^ + 0.708^2^ = 0.925^2^). However, it is clear that the second set of parameters produces a tighter distribution of differences. This is as expected since the intra-method correlations were 0.72 (as above) and 0.883, respectively.

The approach to account for the correlation between two methods, with inter-method correlation of *r*_*yz*_, is to expand the procedure described earlier for combining asymmetric error bars to cover the case where two quantities co-vary. The form of this is:

43 $$\begin{aligned} L = \sqrt {L_{A}^{2} + U_{B}^{2} - 2corr(r_{xy} ,r_{xz} )L_{A} U_{B} } \hfill \\ U = \sqrt {L_{B}^{2} + U_{A}^{2} - 2corr(r_{xy,} r_{xz} )L_{B} U_{A} } \hfill \\ \end{aligned}$$

Here “*corr*()*”* is the correlation between correlation coefficients. To calculate the correlation we first calculate the covariance from *r*_*xy*_, *r*_*xz*_ and *r*_*yz*_. Pearson himself addressed this problem [8]. The approximate formula suggested for cases with a ‘shared’ dataset (in this case *x*), is:

44 $$cov \left( {r_{xy} ,r_{xz} } \right) = \frac{1}{N}\left[ {r_{yz}^{3} + \left( {r_{yz} - 0.5r_{xy} r_{xz} } \right)\left( {1 - r_{yz}^{2} - r_{xy}^{2} - r_{xz}^{2} } \right)} \right]$$

45 $$corr\left( {r_{xy} ,r_{xz} } \right) = \frac{{cov\left( {r_{xy} ,r_{xz} } \right)}}{{\sqrt {var\left( {r_{xy} } \right)var\left( {r_{xz} } \right)} }}$$

The formula for the variance of an *r* value follows from Eq. 44 by setting *z* = *y*, i.e.

46 $$cov\left( {r_{xy} ,r_{xy} } \right) = var\left( {r_{xy} } \right) = \frac{1}{N}\left[ {1 - 2\left( {1 - 0.5r_{xy}^{2} } \right)\left( {r_{xy}^{2} } \right)} \right] = \frac{1}{N}\left[ {1 - r_{xy}^{2} } \right]^{2}$$

Substituting the values for *r*_*xy*_, *r*_*xz*_ and *r*_*yz*_ for the example illustrated in Fig. 6 we compute the following estimates for the 95 % bounds:

1. *r*_*yz*_ = 0.72, corr(*r*_*xy*_, *r*_*xz*_) = 0.36, $\Delta r \in \left[ {0.013, 0.22} \right]$
2. *r*_*yz*_ = 0.883, corr(*r*_*xy*_, *r*_*xz*_) = 0.66, $\Delta r \in \left[ {0.035, 0.205} \right]$

Both estimates are in good agreement with the observed distributions.

If prediction methods are using the same physical principles to predict a quantity of interest they are likely to be “over” correlated. For example, scoring functions used to place and predict binding of ligands to proteins are all really quite similar. They all have contact terms; they all have some variant of an electrostatic interaction term etc. As such, it is likely the differences in any prediction they make are quite correlated. If a prediction method is being developed and version X.1 is being compared to version X.2, where only minor modifications have been made between versions, then any *r* values calculated against the target experimental value are likely over-correlated. It is possible that methods that consider different aspects of a physical phenomenon might be less correlated. If, in fact, the inter-method correlation is less than expected from the product of correlation coefficients then such methods might be more usefully combined.

If the observed difference between two *r* coefficients is greater than the estimate formed by estimating the inter-method covariance from the product of the correlation coefficients then it is likely safe to assert one method has a superior correlation. As with other examples in this paper, correlation between methods can ‘rescue’ significance from examples that do not appear “independently” significant, but if the methods appear significant without the inclusion of correlation this added level of complexity is likely unneeded.

## Thoughts and observations on parametric versus non-parametric modeling of differences

One of the complaints about using classical statistics is that distributions are not always Gaussian. Bootstrapping and ‘non-parametric’ methods do not assume anything about the underlying distribution and hence are more general than many of the approaches described here. They also have the advantage that they can work on lists and rankings, rather than continuous values, and as such clearly have a place in the toolbox of useful statistical methods. For example, Friedman’s test is a non-parametric version of the ANOVA method described later in this paper. Or the Mann–Whitney–Wilcoxon test used to determine if two sets of rankings are different, which is analogous to the tests described above to the paired Student *t* test. Spearman’s *rho* is an analogous correlation statistic to Pearson’s but for ranked lists, etc. [13].

**Fig. 7:**
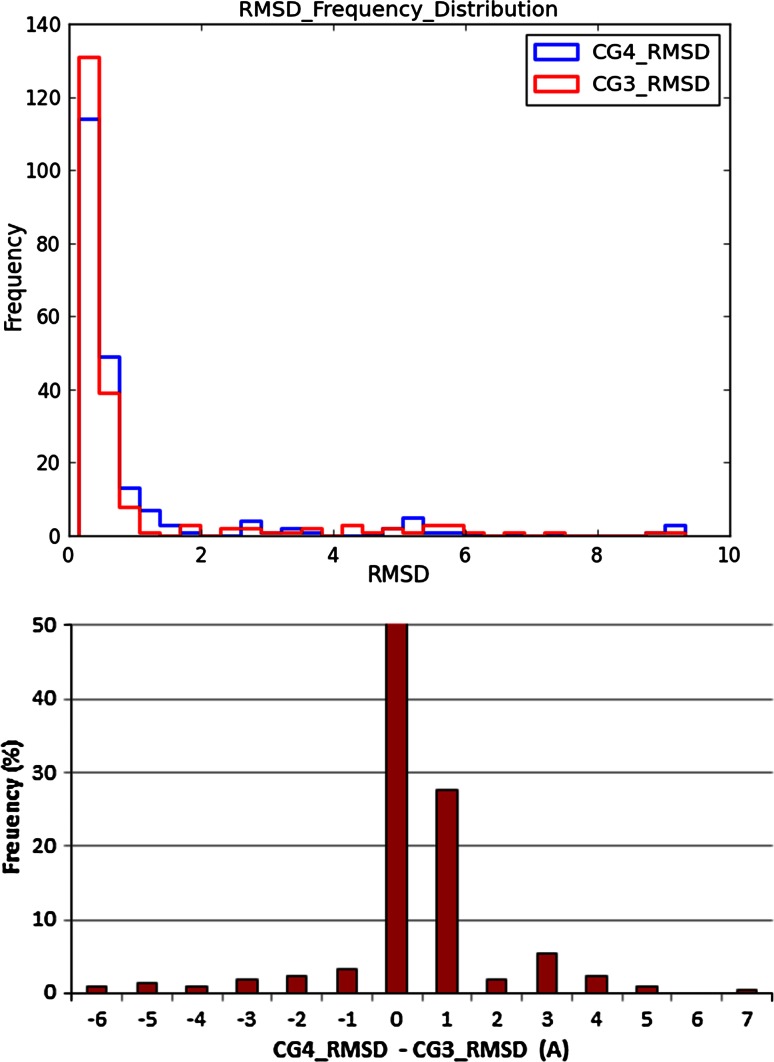
The *top graph* shows the frequency of pose reproduction using two different scoring functions, with RMSD on the *x*-axis and frequency on the *y* axis. The *bottom graph* has on the *x*-axis the difference in RMSD values of paired examples, e.g. the RMSD under CG4 minus the RMSD for CG3 for the same example. Hence this can be negative as well as positive. The *y*-axis is the frequency of observation of this difference

The reason this paper has focused on parametric statistics is: (1) they are more *powerful* than non-parametric statistics, e.g. they give you better, tighter error bounds, (2) for items we are interested in, such as the differences in properties, they are often more robustly applicable than is commonly thought. Consider the two graphs in Fig. 7. The top figure displays the distribution of ligand crystal structure reproductions using a docking program and two scoring functions (CG3 and CG4). On the *x*-axis is the root-mean-square-deviation (RMSD) between heavy atom coordinates of the docked pose and the crystal pose, on the *y* axis the frequency of results. It is clear the distributions in this graph are very non-Gaussian. Furthermore, the average across the distribution is also quite non-Gaussian, i.e. the error bars on the average are very asymmetric. It is for this reason the reporting of a single average RMSD for pose prediction performance is ill advised. The bottom figure displays the frequency of the paired differences between performances of the two scoring functions. While not perfectly Gaussian it is much closer. The variation of the *average* of this difference is distributed accurately as a Gaussian, as the CLT would predict.

A second example would be the difference in *r* values displayed in Fig. 6. Although the distributions of correlation coefficients are quite asymmetric, requiring Fisher’s transformations to regain Gaussian shape, the difference in *r* values is much more normal. Even though underlying distributions may not be very Gaussian, leading some to claim non-parametric statistics or bootstrapping may be more appropriate, *differences* in properties, which is often what interests us, may be well behaved and suitable for the application of standard statistical practices.

## Comparing multiple methods

Often computational chemistry will require the comparison of the performance of multiple methods. Having described how to evaluate a pair of methods it might seem straightforward to evaluate multiple methods-surely this is just a series of pair-wise comparisons? However, multiple-method comparisons can come in several forms:

(i) Compare a select method to a series of other methods
(ii) Determine whether a series of methods are actually equivalent.
(iii) If a set of methods are not equivalent, which are better and by how much?

These are actually quite distinct questions and the complicating factor as to whether the methods are independent or dependent is of great importance for each. Here they will be considered in order.

### Comparing a single method to a series of others

A correct comparison of two methods gives an estimate of the probability that, say, method A is actually better than method B. To accord with standard practice we expect that probability to be greater than 95 %, although this is, of course, arbitrary.

Now, suppose we are comparing method *A* to five different methods, *B* through *F*. Suppose that *A* seems better than each of them, at roughly the same level of superiority, i.e. that there is only a 0.05 chance that one of the other methods is actually better than *A*. What is the probability that A is actually better than all of them? This is equivalent to rolling a twenty sided dice and avoiding a “20” five times, which is:

47 $$p\left( {Zero\;``20''s} \right) = \left( {1 - 0.05} \right)^{5} = 0.773$$

Conversely the probability of at least one “20” occurring is:

48 $$p\left( {One\;or\;More\;``20''s} \right) = 1 - \left( {1 - 0.05} \right)^{5} = 0.227$$

Thus, although the probability of *A* being better than any one alternate method is still *p* = 0.05, the probability of being better than the *group* or *family* of methods, *B* through *F*, is close to five times that at *p* = 0.227. This is referred to as the *Family*-*Wise Error Rate* (FWER) and relates to the probability of making one or more “False Discoveries” (False Positives). This is a topic of considerable interest in fields such as the analysis of correlations between gene sequences or microarray data, where the probability of making a false discovery becomes very high because so many comparisons are considered. In fact, a poor appreciation of this simple phenomenon led many early incorrect predictions of correlations in early genome studies. For instance many gene associations were made to schizophrenia that were later found to be incorrect using Genome Wide Association Studies with more sophisticated meta-analysis [14].

There are several approaches to dealing with this problem. The simplest, as proposed by Bonferroni, is to change what we mean by “significant” [15]. Since the probability of at least one false comparison is roughly proportional to the number of comparisons, *N*, we can regain our *p* = 0.05 sense of (*Family*-*Wise*) significance if we require the threshold for each individual comparison to be reduced *N*–fold, i.e. change our requirement on *p* for an individual comparison to 0.05/5 = 0.01. Doing so returns the probability of making *no* mistakes to ~0.05:

49 $$p\left( {One\;or\;More\;``20''s} \right) = 1 - \left( {1 - 0.01} \right)^{5} = 0.049$$

However, there is a problem with this approach, namely that we may “throw out the baby with the bathwater”. In trying to avoid *any* false positive mistakes, we might make *false negative* ones, i.e. suppose method *B* has a probability of being better than *A* of 0.02 and the *pvalues* of *C* through *F* are all really low, say *p* = 0.0001. Because we have included a number of really poor methods in the comparison, suddenly *B* doesn’t look so bad, i.e. because *p* = 0.02 > *p* = 0.05/5 = 0.01. This can lead to confusion—how can adding bad methods suddenly make a method such as *B* seem significant?

Remarkably, a very simple method can address this conundrum: *step*-*wise* Holm–Bonferroni [16]. It is a very simple method.

(i) Order methods (*B*, *C*, *D*, *E*, *F*) such that the method least likely to actually be better than *A* is first, i.e. the one with the smallest *p* value, with the one most likely to be better than *A* last, i.e. the one with the largest *p* value.
(ii) Test the first method against not *p* = 0.05 but *p* = 0.05/(*N* + 1 − *k*) where *N* is the number of methods (*N* = 5) and *k* is the position in the list (*k* = 1).
(iii) If *A* passes this test (i.e. *p*_1_ < 0.05/(5 + 1 − 1) = 0.01), move on to the next method, adjusting *k* upwards by one.
(iv) If *A* passes all the comparisons, the probability it is actually better than all methods is better than *p* = 0.05.
(v) If it fails a test against a method in the list, assume that this alternative method and all the methods with larger *p* values are also failures.

In our above example, method *B* would be at the end of the list, i.e. is only compared against *p* = 0.05. As such, it is always seems insignificant with respect to *A*, i.e. equivalent, no matter if much worse methods (with much lower *p* values) are ahead of it in the list. As a more concrete example, suppose we have two possible lists of *p* values for B through F as in Table 1.

**Table 1:** Two example sets of example *p* values for a ‘family’ of five methods versus a sixth method, *A*, i.e. the *p* value represents the probability any of these methods could actually be better than *A* even though their mean performance is worse

Method	List 1	List 2
B	0.02	0.01
C	0.005	0.025
D	0.01	0.005
E	0.03	0.03
F	0.008	0.015

For the “List 1” the procedure gives:

50 $$\begin{aligned}& \{ B,C,D,E,F) \to \left\{ {C,F,D,B,E} \right\} \hfill \\& p_{C} < \frac{0.05}{5};\quad p_{F} < \frac{0.05}{4};\quad p_{D} < \frac{0.05}{3};\quad p_{B} < \frac{0.05}{2};\quad p_{E} < 0.05 \hfill \\ \end{aligned}$$

Therefore, the probability that method *A* is better than all five methods, with no misdiagnosis is less than 0.05 (the FWER is less than 0.05).

For “List 2”:

51 $$\begin{aligned} \{ B,C,D,E,F) \to \left\{ {D,B,F,C,E} \right\} \hfill \\ p_{D} < \frac{0.05}{5};\quad p_{B} < \frac{0.05}{4};\quad p_{F} < \frac{0.05}{3};\quad p_{C} = \frac{0.05}{2} \hfill \\ \end{aligned}$$

Therefore, we should accept that *A* is better than methods *D*, *B* and *F* but not methods *C* and *E*, because the Holm–Bonferroni test tells us there is a greater than 0.05 chance that either *C* or *E* are not actually significantly better that *A* at the *p* = 0.05 confidence level.

Does it matter if methods are correlated or not? At first glance it might seem irrelevant. If we have accounted for the correlation between method *A* and each of the methods in the list, i.e. so we obtain a correlation-corrected *pvalue* then what else would we need? Does it matter if methods *B* to *F* are correlated with each other? In general, a useful way to think about the effects of correlation is to imagine two methods are essentially identical, i.e. perfectly correlated. For instance, suppose methods *C* and *D* are actually the same method. We have gained nothing by adding both rather than just one, i.e. no new information. However, the total number of methods considered has been increased by one if both are included. In the Holm–Bonferroni method this means that the criteria for being declared significant have been made harder than necessary, i.e. we only had to check against four methods with less stringent testing because we could have omitted *C* or *D*. As such, Holm–Bonferroni is *conservative* with respect to correlation effects, i.e. if something is declared significant we can trust it was tested at the appropriate level, but we may have dismissed something as insignificant that is not.

Typically a ‘conservative’ method means there are other methods that are less conservative (more likely to make false positive mistakes) but which have more “power”, i.e. can resolve true positives. The Holm–Bonferroni is no exception and there is a different method from Hochberg et al. that is a *step*-*up* procedure [17]. In this method we start at the other end of the list, progressing until a test is *passed*. At this point the rest of the methods are assumed different from the primary method. This has more resolving power, i.e. will correctly assess more differences in performance. However it makes more assumptions as to the independence of methods.

It could be that we do not care as to whether method *A* is better than *all* other methods, perhaps we are happy with just knowing it is likely to be better than *most* methods. What we might be interested in is an estimate of how many false positives we have likely included by mistake. For instance, in a virtual screening list it would be very useful to say how many ligands in our top 100 are likely inactive. This problem falls under the rubric of control of the False Discovery Rate (FDR), as developed by Benjamini, Hochberg and others [18].

### Determine whether a series of methods are actually equivalent to each other

In the above section we considered the case of a binary categorization, up or down, yes or no, and the simple formula that allows us to know if the difference in choices was significant. An obvious generalization is when the number of groups is greater than two. This has several potential uses, for instance, we could be comparing methods that produce categorizations over a number of examples. Perhaps the categories are “antagonist”, “agonist”, “inactive” for some GPCR system, or perhaps they are some ordinal ranking scheme, e.g. “1” to “5” for some property.

Suppose we have *N* examples to be classified into *M* categories where method *A* distributes values as *E*_*i*_ whereas method *B* distributes them according to *F*_*i*_ : are the results different? Our “NULL” hypothesis is that they are not different, therefore the question is whether we think either method is a reasonable deviation from the frequencies expected in each category. Consider the following sum over categories:

52 $$\chi^{2} = \mathop \sum \limits_{i = 1}^{M} \frac{{\left( {E_{i} - \left( {E_{i} + F_{i} } \right)/2} \right)^{2} }}{{\left( {E_{i} + F_{i} } \right)/2}} = \mathop \sum \limits_{i = 1}^{M} \frac{{\left( {F_{i} - \left( {E_{i} + F_{i} } \right)/2} \right)^{2} }}{{\left( {E_{i} + F_{i} } \right)/2}}$$

I.e. we are dividing the square of the deviation from the expected frequency by the expected frequency (formed from the average of the two methods). We label this sum χ^2^ because Pearson showed it is equivalent to the Chi squared function described in Part One, i.e. the expected sum of the *square* of random draws from a unit Gaussian [19]. E.g. if you select *N* random numbers from a Gaussian of unit width, square each number and add them together you obtain a sum distributed as the Chi squared function. We can develop a sense of why this must be true for Eq. 52 by rewriting it in terms of observed class probabilities, *p*, relative to the expect probability, *P*.

53 $$\chi^{2} = \mathop \sum \limits_{i = 1}^{M} \frac{{N\left( {p_{i} - P_{i} } \right)^{2} }}{{P_{i} }}$$

We should note that since the sum of probabilities is constrained to one, there is one less degree of freedom that in the sum, i.e. *M* − 1, i.e. just as we use (1*/N* − 1) rather than (1*/N*) in calculating variances we should scale this sum by (*M* − 1*/M*). If we recall that the variance for a probability is *P*(1 − *P*) then this looks like:

54 $$\chi^{2} = \mathop \sum \limits_{i = 1}^{M} \frac{{N\left( {\Delta p_{i} } \right)^{2} }}{{Var_{i} }} = \mathop \sum \limits_{i = 1}^{M} \left( {\sqrt {\frac{{\Delta p_{i} }}{{Var_{i} /N}}} } \right)^{2}$$

Each term here looks like a square of a *t* statistic for the deviation of the frequency of observations for that category from the expected, i.e. the sum looks like the Chi-squared function. The degrees of freedom of that function (i.e. the number of draws) is not *M* here but (*M* − 1) because the probabilities have to add up to one, i.e. there is one less degree of freedom than we expect. This is taken care of by the fact that we have treated the variance as if it is just *p* not *p*(1 − *p*), i.e. each denominator is actually larger than it should be, reducing the sum by, on average (*M* − 1)/*M*.

This, then, is Pearson’s Chi-squared test for two methods of categorization. If, instead, we are comparing a single method to a set of standard frequencies, *O*_*i*_, we derive:

55 $$\chi^{2} = \mathop \sum \limits_{i = 1}^{M} \frac{{\left( {E_{i} - O_{i} } \right)^{2} }}{{O_{i} }}$$

If we consider there being only two categories, returning to the earlier binary example, the Chi-squared statistic is:

56 $$\begin{aligned}& \chi^{2} = \mathop \sum \limits_{i = 1}^{2} \frac{{\left( {E_{i} - N/2} \right)^{2} }}{N/2} = \mathop \sum \limits_{i = 1}^{2} \frac{{\left( {\Delta N/2} \right)^{2} }}{N/2} \hfill \\& N\chi^{2} = \left( {\Delta N} \right)^{2} \hfill \\ \end{aligned}$$

Here we have only one degree of freedom (the difference) and the Chi-squared value for 95 % significance is 3.84 (1.96 × 1.96) ~ 4.0, i.e. leads to an identical (asymptotic) formula as in Eq. 20 in the previous section.

57 $$\left( {\Delta N} \right)^{2} > 4N$$

Chi-squared is reintroduced here because it plays a central role in the evaluation of the significance of groups of methods, as it does in Pearson’s test for categories. This is especially clear in the derivation of Fisher’s *F* function that lies at the center of both ANOVA and, more generally, the comparison of “nested” models.

A “nested” model is simply one that is a more elaborate version of a simpler one, e.g. if we go from a one-parameter model to a two-parameter model then the second model is said to be “nested” relative to the first. ANOVA is Fisher’s classic technique to determine if a technique (method) is having an effect relative to a set of other techniques (methods) [20]. For instance, you may have a large population where each member has a set of characteristics (e.g. smoker, vegan, gender, race). If you have some outcome (e.g. disease prevalence) you can try to separate out the population into sub-populations where only one category varies and then attempt to say whether that category is correlated with the outcome. Use of ANOVA and its many variants is widespread in the medical and social sciences and, initially, seems quite removed from whether two models are equivalent. As a consequence, at least in this author’s experience, ANOVA, while simple to perform, is conceptually confusing. Here, the basics are described in a standard manner but are followed by a simpler and more powerful explanation as to why ANOVA works, and how it can be extended.

In ANOVA the starting assumption is that everything is the same, e.g. there is no difference in the subpopulations with different characteristics. Then, two “sums of squares” are calculated. The first is the sum of the squared differences of the average of each subpopulation from the “global” mean. This measures the “variance” of the subpopulations’ averages from the mean of the entire population, multiplied by the number of examples in the subset. The second “sum of squares” is the sum of the squared differences of each member of the population from its “local” subpopulation mean. (Note that the terms used here of “local” and “global” are not usual, rather terms such as “between groups” and “within groups”, respectively, are often used). Formally, if there are *M* subpopulations, each with a population of *N*, then we have for the two key sum-of-squares:

58 $$\begin{aligned} SS_{global} = & N\mathop \sum \limits_{i = 1}^{M} \left( {\left\langle x \right\rangle_{i}^{local} - \left\langle x \right\rangle^{global} } \right)^{2} \\ SS_{local} = & \mathop \sum \limits_{i}^{M} \mathop \sum \limits_{j = 1}^{N} \left( {x_{j} - \left\langle x \right\rangle_{i}^{local} } \right)^{2} \\ \end{aligned}$$

Note, there is no need for each population to have the same size, rather this has been chosen for simplicity and for easy translation to the case where methods are being compared to the same set of *N* datasets.

Each of these is a sum of squares of quantities drawn from Gaussian distributions. The Gaussian width of the “global” term will be smaller than that for the “local” term because it is drawing from the Gaussian distribution of an average for a set of *N* methods, i.e. each term in the global term is the difference of averages. But we know this width will be smaller by exactly √N, because that is how many items there are in each average. Thus, multiplying the sum of such *squared* quantities by *N* makes up for this and then both terms become draws from equivalent Gaussians (i.e. Gaussians of the same width). In fact, if we divide each term by the number of degrees of freedom of each sum then they ought to be comparable, i.e. close to 1.0 if the methods are equivalent to each other.

Mathematically the ratio of the two sums-of-squares looks like the ratio of two Chi-squared functions. This is precisely how Fisher’s *F* function is constructed, i.e.

59 $$F\left( {i,j} \right) = \frac{{\chi_{i}^{2} /i}}{{\chi_{j}^{2} /j}}$$

If the methods are actually distinct, i.e. have different means, then the term in the denominator will be smaller than expected and *F*(*i*,*j*) will be larger than one. The more extreme the deviation the larger *F* becomes. As the Chi-squared function represents a distribution, i.e. a probability of a sum of squares sampled from a unit Gaussian, so does *F*. As such, we can say how much of the distribution (probability) lies beyond a certain value, i.e. we can tabulate ‘significance’ values, i.e. the likelihood that methods that are actually the same appear to be different. This is a little more difficult than with the Student *t* test because now there are two numbers, *i* and *j*, not just one, i.e. the sample size.

As a worked example, suppose we have three methods and their AUC scores across five protein systems, as in Table 2 below:

**Table 2:** An example of three methods and their performance, e.g. an AUC value, measured over a set of five systems

System	Method A	Method B	Method C
1	0.60	0.81	0.74
2	0.65	0.75	0.7
3	0.7	0.72	0.85
4	0.45	0.69	0.7
5	0.5	0.8	0.75
Average	0.580	0.754	0.748

It looks like method *A* is not as good as the other two. The average performance across all systems is 0.694, and the sum of squares of these method’s average scores from this “global” average:

60 $$SS_{global} = 5 \times [\left( {0.580 - 0.694} \right)^{2} + \left( {0.754 - 0.694} \right)^{2} + \left( {0.748 - 0.694} \right)^{2} ] = 0.0998$$

The “local” sum of squares of method *A* is:

61 $$SS\left( A \right) = \left( {0.6 - 0.58} \right)^{2} + \left( {0.65 - 0.58} \right)^{2} + \left( {0.7 - 0.58} \right)^{2} + \left( {0.45 - 0.58} \right)^{2} + \left( {0.5 - 0.58} \right)^{2} = 0.044$$

For methods *B* and *C*, we have:

62 $$SS\left( B \right) = 0.01;\quad SS\left( C \right) = 0.0152$$

The total local sum-of-squares is then:

63 $$SS_{local} = SS\left( A \right) + SS\left( B \right) + SS\left( C \right) = 0.0696$$

Next we calculate the numbers of degrees of freedom for both sums-of-squares. For the global term we have three terms, each of which share a mean derived from them, i.e. there are two degrees of freedom (i.e. one of the method averages could be replaced using the mean and the other two values). For the local sum there are fifteen squares but we use three means, so there are twelve degrees of freedom. This gives us an *F* value of:

64 $$F = \frac{0.0998/2}{0.0696/12} = 8.45$$

If the methods were all essentially the same we would expect *F* to be close to 1.0. As it is greater than 1.0 we need to know whether a value of 8.45 would be likely, i.e. how likely would it be to see such inter-method (global) variance relative to the intra-method (local) variance that we observe. For this we have to look at the level of significance associated with the critical value for *F*(2,12) value, i.e. the ratio of Chi-squared functions with two and twelve degrees of freedom. The 95 % confidence value for *F*(2,12) is 3.88 so clearly we can be quite confident in believing the methods are not equivalent. In fact, the probability of seeing such a greater degree of variability of the method mean values, given the variability within the methods, is less than *p* = 0.01.

Suppose there are just two methods, *A* and *B*, how does ANOVA then look?

65 $$\begin{aligned} SS_{global} = & N\left( {\left\langle A \right\rangle - \frac{\left\langle A \right\rangle + \left\langle B \right\rangle }{2}} \right)^{2} + N\left( {\left\langle B \right\rangle - \frac{\left\langle A \right\rangle + \left\langle B \right\rangle }{2}} \right)^{2} = \frac{N}{2}\left( {\left\langle A \right\rangle - \left\langle B \right\rangle } \right)^{2} \\ SS_{local} = & \mathop \sum \limits_{i = 1}^{N} \left( {x_{i}^{A} - \left\langle A \right\rangle } \right)^{2} + \mathop \sum \limits_{i = 1}^{N} \left( {x_{i}^{B} - \left\langle B \right\rangle } \right)^{2} = \left( {N - 1} \right)\left( {\sigma_{A}^{2} + \sigma_{B}^{2} } \right) \\ \end{aligned}$$

The number of degrees of freedom for the global term is one, for the local term it is (N − 1) × 2. Therefore the *F* factor is:

66 $$F = \frac{{N\left( {\left\langle A \right\rangle - \left\langle B \right\rangle } \right)^{2} /2}}{{\left( {N - 1} \right)\left( {\sigma_{A}^{2} + \sigma_{B}^{2} } \right)/\left( {\left( {N - 1} \right)*2} \right)}} = \frac{{N\left( {\left\langle A \right\rangle - \left\langle B \right\rangle } \right)^{2} }}{{\left( {\sigma_{A}^{2} + \sigma_{B}^{2} } \right)}}$$

This is exactly the square of the *t* statistic for the paired-*t* test. In fact, given that the function *F*(1,*N*) is the square of the Student *t* function this shows that the two methods are exactly equivalent when comparing just two systems.

#### Alternate interpretation of ANOVA in terms of model comparison

There is an alternate form of ANOVA that is appealing because it extends the concept to a much wider range of problems [21]. Rather than presenting the perhaps mysterious “between-groups” and “within-groups” concept, suppose we looked at the two sums-of-squares as different models for explaining the total variance across all examples. Rather than the “between-groups”, let’s consider a third sum-of-squares, namely the “total” sum-of-squares, i.e. a sum of each of *N* systems and each *M* models:

67 $$SS_{total} = \mathop \sum \limits_{i = 1}^{M} \mathop \sum \limits_{j = 1}^{N} \left( {x_{j}^{i} - \left\langle x \right\rangle } \right)^{2}$$

We would normally refer to this as (3*N* − 1)*Var_*total*_, i.e. it’s an estimate of the variance using all the examples and the global mean. What is noteworthy is that:

68 $$SS_{total} = SS_{global} + SS_{local}$$

This follows from:

69 $$\begin{aligned} SS_{total} = & \mathop \sum \limits_{i = 1}^{M} \mathop \sum \limits_{j = 1}^{N} \left( {x_{j}^{i} - \left\langle x \right\rangle^{global} } \right)^{2} \\ = & \mathop \sum \limits_{i = 1}^{M} \mathop \sum \limits_{j = 1}^{N} \left( {x_{j}^{i} - \left\langle x \right\rangle_{i}^{local} + \left\langle x \right\rangle_{i}^{local} - \left\langle x \right\rangle^{global} } \right)^{2} \\ = & \mathop \sum \limits_{i = 1}^{M} \mathop \sum \limits_{j = 1}^{N} \left( {x_{j}^{i} - \left\langle x \right\rangle_{i}^{local} } \right)^{2} + \mathop \sum \limits_{i = 1}^{M} \mathop \sum \limits_{j = 1}^{N} \left( {\left\langle x \right\rangle_{i}^{local} - \left\langle x \right\rangle^{global} } \right)^{2} \\ \quad - 2\mathop \sum \limits_{i = 1}^{M} \mathop \sum \limits_{j = 1}^{N} \left( {x_{j}^{i} - \left\langle x \right\rangle_{i}^{local} )(\left\langle x \right\rangle_{i}^{local} - \left\langle x \right\rangle^{global} } \right) = SS_{local} + SS_{global} \\ \end{aligned}$$

The final equivalence arises because the third term sum must be zero. We can also derive that the degrees of freedom for the total term has to equal that for the global and local terms, e.g.

70 $$df\left( {total} \right) = NM - 1 = M - 1 + M\left( {N - 1} \right) = df\left( {global} \right) + df\left( {local} \right)$$

Given this identity we can change the *F* statistic to look like:

71 $$F = \frac{{SS_{global} /df_{global} }}{{SS_{local} /df_{local} }} = \frac{{\left( {SS_{total} - SS_{local} } \right)/\left( {df_{total} - df_{local} } \right)}}{{SS_{local} /df_{local} }}$$

We can look at the RHS of the equation in a completely new way. We can view the numerator as the improvement in the “error” in a model that uses a single number (the global mean) to predict all the values in the set, as opposed to having a different mean for each method. The *F* function becomes a measure of *model improvement*. In this guise it is a much more powerful entity and is know as the *Ftest* for nested models (as here), i.e. where we add explanatory parameters and look to see whether the improvement in the sum of the errors (squared) is large enough, in a relative sense, to justify a more complex model. ANOVA is just one example of an *F test* where the simple model has a single mean (all methods are equivalent) versus a separate mean for each grouping (each model is distinct).

One of the advantages of looking at ANOVA in this way is that it is then straightforward to think of variations. For instance, suppose we want to test whether a subset of methods is equivalent but the remainder are different—remember ANOVA actually tests whether *all* the methods are different from each other. Here the *SS*_*total*_ term would remain the same but the *SS*_*local*_ term would use the global mean for each method in the subset we assume equivalent. We would then adjust the degrees of freedom, *df*_*local*_, to reflect how many different means are present in the *SS*_*local*_ term, calculate *F* and compare it to the critical value of interest for *F*(*df*_*global*_, − *df*_*local*_, *df*_*local*_).

What is great about the *F* test is that as long as we expect the errors in a prediction method to be distributed in a Gaussian manner it is a quite general test to control for parameterization in nested models. All we have to do is to replace the sums of squares with those produced in making predictions with the two models being compared. Although outside the scope of this article, analytic approaches for assessing over-parameterization, such as the Fisher *F* test, are very attractive as an alternative to cross-validation or bootstrapping, for instance Rivals and Personnaz [22]. A similar example exists for Pearson’s *r*^2^, known as the adjusted *r*^2^, is defined as [23]:

72 $$\overline{{r^{2} }} = r^{2} - \left( {1 - r^{2} } \right)\frac{p}{N - p - 1}$$

Here *N* is, as usual, the number of data points and *p* is the number of parameters introduced into the model to fit the data. Hence, the second term will tend to reduce *r*^2^. This is appropriate as more parameters may improve the *apparentr* value without actually adding predictive power. Thus adjusted *r*^2^ values are frequently used to compare models with different numbers of fitting parameters. Note, however, that Romero points out that this construct lacks statistical analysis of *risk*, i.e. the discernment of how often you will be led astray into thinking one *r*^2^ is actually greater than another. In fact, he recommends the use of an *F* test! [24].

#### Correlated methods

When ANOVA is used in medical, economic or social sciences it is typically applied to groupings of people, social strata, countries, etc. In these cases each example is a distinct entity, i.e. someone who smokes and is a vegan is not also a smoker and a carnivore. However, the examples under study in comparing models in computational chemistry are often the exact same entities under multiple treatments. For instance, in the example in Table 2 with three methods and five systems, the exact same five systems were used to evaluate each model. The assumptions underlying the elegant mathematics of the *F* test are that the sums-of-squares we calculate in the local and global sums are *independent* draws from unit Gaussians. But this is far from guaranteed, as we have seen in considering the comparison of a pairs of methods. As such, we have to take care if we want to obtain good estimates of significance, which include consideration of correlation between methods. As with the Holm–Bonferroni adaption to successive significance testing, ignoring correlation typically means that tests become more conservative than necessary, i.e. if something still seems significant under the assumption of independence they will be so under more exacting consideration of correlation. However, the reverse may not be true, i.e. if your test says things are the same and assumes independence that may not be true when correlation is included.

Dealing with correlation for multi-method comparison is conceptually more difficult, and more mathematically involved. The key concept is the idea that a linear transformation of correlated random variables can bring us back to a set of random variables that now act as uncorrelated, i.e. independent. Tests meant for the original variables are now applied to the transformed variables. If such a test suggests that these transformed variables come from the same distribution, e.g. are equivalent, then so are the original variables. The key concept is how to generate the independent variables. This revolves around knowing the *covariance* matrix, i.e. the matrix with element (*i*,*j*) equal to the covariance of method *i* and *j*. This is a symmetric matrix, i.e. *covar*(*i*,*j*) = *covar*(*j*,*i*) and a linear transformation will diagonalize this matrix with real eigenvalues. As an example of a three method system:

73 $$V = covar\left( {x_{3x3} } \right) = \frac{1}{N - 1}\left[ {\begin{array}{*{20}c} {\overrightarrow {{x_{1} }} } \\ {\overrightarrow {{x_{2} }} } \\ {\overrightarrow {{x_{3} }} } \\ \end{array} } \right].\left[ {\begin{array}{*{20}c} {\overrightarrow {{x_{1} }} } & {\overrightarrow {{x_{2} }} } & {\overrightarrow {{x_{3} }} } \\ \end{array} } \right]$$

Where each entry, *x*, is a vector of results over M systems, e.g.

$$\overrightarrow {{x_{1} }} = \left( {x_{1,1} - \left\langle x \right\rangle_{1} ,x_{1,2} - \left\langle x \right\rangle_{2} , \ldots ,x_{1,M} - \left\langle x \right\rangle_{M} } \right)$$

74 $$\begin{aligned} V = & \left[ {\begin{array}{*{20}c} {var\left( {1,1} \right)} & {covar\left( {1,2} \right)} & {covar\left( {1,3} \right)} \\ {covar\left( {2,1} \right)} & {var\left( {2,2} \right)} & {covar\left( {2,3} \right)} \\ {covar\left( {3,1} \right)} & {covar\left( {3,2} \right)} & {var\left( {3,3} \right)} \\ \end{array} } \right] \\ = & \left[ {\begin{array}{*{20}c} {a_{11} } & {a_{12} } & {a_{13} } \\ {a_{21} } & {a_{22} } & {a_{23} } \\ {a_{31} } & {a_{32} } & {a_{33} } \\ \end{array} } \right]\left[ {\begin{array}{*{20}c} {\lambda_{1}^{2} } & 0 & 0 \\ 0 & {\lambda_{2}^{2} } & 0 \\ 0 & 0 & {\lambda_{3}^{2} } \\ \end{array} } \right]\left[ {\begin{array}{*{20}c} {a_{11} } & {a_{21} } & {a_{31} } \\ {a_{12} } & {a_{22} } & {a_{32} } \\ {a_{13} } & {a_{23} } & {a_{33} } \\ \end{array} } \right] = A\varLambda^{2} A^{T} \\ \end{aligned}$$

The matrix *A* is a generalized rotation matrix, i.e. its transpose is its inverse. Here, the eigenvalues have been written as squares because we know they are positive and because it allows us to define the inverse square root of this matrix:

75 $$V^{ - 1/2} = A\left[ {\begin{array}{*{20}c} {\frac{1}{{\lambda_{1} }}} & 0 & 0 \\ 0 & {\frac{1}{{\lambda_{2} }}} & 0 \\ 0 & 0 & {\frac{1}{{\lambda_{3} }}} \\ \end{array} } \right]A^{T} = W$$

It should be easy to see that, given the rotation matrix *A* is orthogonal to its transpose, that *W*^2^ gives us the inverse of the covariance matrix, *V*^−1^. As such, *W* forms a transformation that combines the three methods into a new set of three methods, *y*_1_, *y*_2_ and *y*_3_, that are independent of each other and each have unit variance.

76 $$WVW^{T} = \frac{1}{N - 1}W\left[ {\begin{array}{*{20}c} {\overrightarrow {{x_{1} }} } \\ {\overrightarrow {{x_{2} }} } \\ {\overrightarrow {{x_{3} }} } \\ \end{array} } \right].\left[ {\begin{array}{*{20}c} {\overrightarrow {{x_{1} }} } & {\overrightarrow {{x_{2} }} } & {\overrightarrow {{x_{3} }} } \\ \end{array} } \right]W^{T} = \frac{1}{N - 1}\left[ {\begin{array}{*{20}c} {\overrightarrow {{y_{1} }} } \\ {\overrightarrow {{y_{2} }} } \\ {\overrightarrow {{y_{3} }} } \\ \end{array} } \right].\left[ {\begin{array}{*{20}c} {\overrightarrow {{y_{1} }} } & {\overrightarrow {{y_{2} }} } & {\overrightarrow {{y_{3} }} } \\ \end{array} } \right] = I$$

Therefore, in this new “coordinate” frame, we can calculate a Chi-squared statistic with *M* − 1 degrees of freedom that can be used in ANOVA. The form of this sum in the transformed “coordinates” is:

77 $$\chi^{2} = \left( {\overrightarrow {\left\langle x \right\rangle } - \mu \vec{1}} \right)^{T} W^{T} \cdot W \cdot \left( {\overrightarrow {\left\langle x \right\rangle } - \mu \vec{1}} \right)$$

Where the *x* vector is the vector of averages for each method, 1 is a vector of “1”s and *μ* is the scalar mean that minimizes this (Chi-squared) expression. It is straightforward to show that

78 $$\mu = \left( {\overrightarrow {{1^{T} }} V^{ - 1} \vec{1}} \right)^{ - 1} \overrightarrow {{1^{T} }} \cdot V^{ - 1} \cdot \overrightarrow {\left\langle x \right\rangle }$$

For this value of *μ* Chi-squared takes the form:

79 $$\chi^{2} = \left( {\overrightarrow {{\left\langle x \right\rangle^{T} }} V^{ - 1} \overrightarrow {\left\langle x \right\rangle } } \right) - \left( {\overrightarrow {{\left\langle x \right\rangle^{T} }} V^{ - 1} \vec{1}\left( {\overrightarrow {{1^{T} }} V^{ - 1} \vec{1}} \right)^{ - 1} \overrightarrow {{1^{T} }} V^{ - 1} \overrightarrow {\left\langle x \right\rangle } } \right)$$

The first term here replaces the sum of *x*^2^ in a uncorrelated Chi-squared and the second term replaces the square of the mean of these terms, divided by *M*, the number of methods. This is equivalent to the *SS*_*global*_ term in ANOVA. We can also calculate the equivalent of the “local” ANOVA term using the “local” averages of each transformed method.

### Distinguishing single methods from a set of methods

If we run ANOVA, either on uncorrelated or correlated data, we might determine that all the methods are not equivalent, i.e. one or more are statistically better or worse. How to we prove this is the case for a particular method?

If the methods are uncorrelated then we can follow the prescription of Holm and Bonferroni, i.e. we can test whether a particular method is better than the others by assessing if it is significant *enough* in a step-down method. In fact, if the methods are independent one can adopt a slightly more powerful *step*-*up* procedure due to Hochberg.

For completeness, the Honest Significance Difference (HSD) method of Tukey should also be mentioned [25, 26]. This approach involves its own special function that measures the expected maximum separation between methods drawn from a Student *t*-function of a given number of degrees of freedom. Each difference between two methods average performance is then turned into a “*t*-like” statistic by dividing by a pooled estimate of the standard deviation of this average, e.g.

80 $$t_{HSD} \left( {i,j} \right) = \frac{{\left| {\left\langle i \right\rangle - \left\langle j \right\rangle } \right|}}{{\sqrt {\mathop \sum \nolimits_{i = 1}^{M} var_{i} /\left( {N \times M} \right)} }}$$

As an example, for the three methods, *A*, *B* and *C* described in Table 2, we obtain a pooled standard deviation (the denominator of Eq. 80) of 0.034 (*N* = 5, *M* = 3). The value of the HSD function for three methods, each with five examples at 95 % significance is 3.77 (note, tabulated versions of this function are not easy to find!). This is then compared to the HSD *t* values for the difference between each pair of methods, e.g.

81 $$t_{HSD}^{{\left| {A - B} \right|}} = 5.12; \quad t_{HSD}^{{\left| {A - C} \right|}} = 4.94; \quad t_{HSD}^{{\left| {B - C} \right|}} = 0.18;$$

Clearly, as expected, *A* is significantly different from *B* and *C* (*t*_HSD_ > 3.77), whereas *B* and *C* are statistically similar (*t*_HSD_ < 3.77).

If the methods are correlated then the situation is more demanding. However, we have all the tools necessary from the preceding sections. First, extract out the method of interest. Second, for the remaining methods, run the procedure above for constructing uncorrelated linear superpositions for methods. Let’s assume that the Chi-squared test on this remainder set show that they do not show significantly different behavior. We can then test our chosen method against each of the linear superposition of methods for significance, following the Holm–Bonferroni step-down approach or the Hochberg step-up approach (the latter would be preferred as we know the methods being tested *against* are now independent). Note that the superpositions of (equivalent) methods *will* be correlated with our chosen method; it is just that they are not correlated within themselves that matters.

If more than one method is to be tested then we can follow a hybrid approach, i.e. we can adapt ANOVA as described above to test whether sets of methods are better described by a single mean or individual means, via an *F* test. The situation is, of course, more complicated when methods are correlated. However there is nothing to stop us developing subsets such that each are made into linear superpositions of methods. Such an approach then “blocks” of methods that are statistically equivalent, but which are statistically different from other blocks. These methods follow fairly naturally from the above exposition but the interested reader is directed for more details to what is called *Structural Equation Modeling* (SEM). SEM considers such ‘block’ significance with much more rigor than can be provided here [28].

#### Depiction of statistical significance of sets of methods

When a paper is written to compare different methods it is quite typical to give a histogram representing the performance of each methods, where each bar is decorated with error bars. However, such depictions do not show the inter-correlations between methods that, as we have seen, can affect how the differences between performances ought to be viewed. One solution is to present the complete matrix of inter-method difference along with associated error bars on such differences. Although such data reporting is important for completeness it is sparse in its interesting content. For instance, if method A is significantly better than method B, and method B is significantly than method C then it is unlikely that method A is worse than method C.

**Fig. 8:**
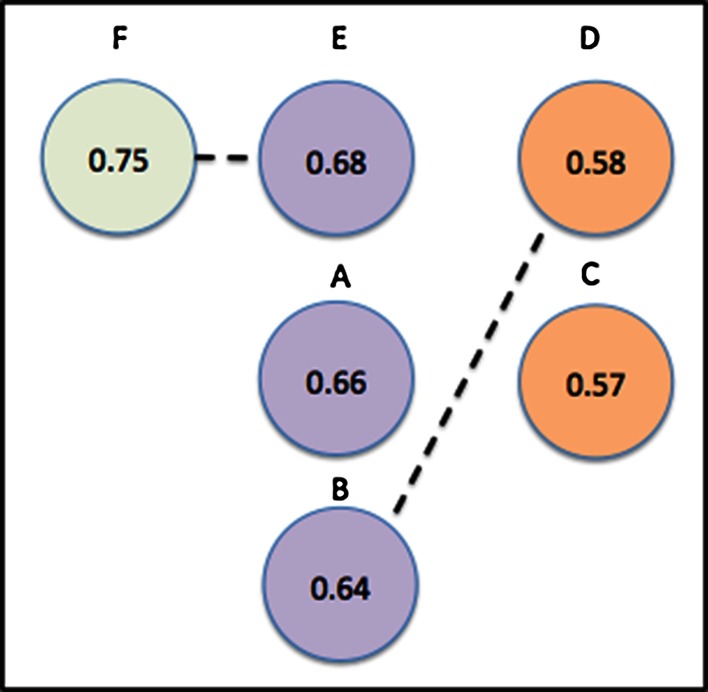
A suggestion as to how to represent multiple method performance where the statistical relevance of relative performance can be made obvious

What should be inherently of interest is harder to discern from either a traditional array of histogram bars or from a matrix of method correspondences. For instance, we would like to know what blocks of methods are essentially equivalent, and which are better than others. Figure 8 presents a suggestion for the depiction of the performance of a set of methods, in this case *A* through *F*. The rules for generating this depiction are simple. Methods are represented by circles, inscribed with their average performance (optionally with 95 % ranges of that performance). Methods that are statistically indistinguishable from each other are put in the same column, ordered within that column from top (best) to bottom (worse). The columns are ordered from left (best) to right (worse) based on the performance of the method at the top of the column. Methods that are statistically equivalent *between* columns are joined by a dotted line (e.g. see *F* and *E*, *B* and *D* below).

If, for instance, all methods are statistically equivalent then this reduces to a single column ordered from best to worse, top to bottom. Figure 8 illustrates a more interesting example. Methods *E*, *A* and *B* form a grouping (the difference between any pair in this set is insignificant at 95 % confidence), as do *D* and *C*. Although method *F* is statistically better than methods *A*, *B*, *D* and *C*, is it not different from method *E*. Similarly, methods *B* and *D* are statistically similar, even though *B* stands out from *C*.

## Discussion of conceptual issues concerning confident intervals and significance testing

The idea of a *pvalue* is that it tries to summarize how likely it is that something we thought we observed was in fact due to chance. For instance, we made a new scoring function and tried it out on a few of our favorite systems and it seemed to do better than our old one. Is this real or due to random chance? This was the central plank of Fisher’s efforts to make statistics “scientific”, i.e. provide a firm foundation for determining what we actually know during research.

However, it turns out that *pvalues* have real problems, so much so that there are periodic pushes in some fields to abandon them from publications [28]. One of the issues, as discussed below, is that they are not an intrinsic aspect of a system, or pair of systems, but an extrinsic property, i.e. the more measurements on a system the smaller a *pvalue* can be made. Any drug can be made significant at a *p* = 0.05 level if the clinical trial is made large enough (although not necessarily significantly *better*).

Another concern is that *pvalues* are often misunderstood. Suppose we have designed a blood test for exposure to a certain virus and we set the threshold for “significance” at 95 %, i.e. if someone has not been exposed (the “NULL” model) then the chance of exceeding this threshold due to random variation is 5 %. Suppose you now take that test and come up positive. What is the probability you have been exposed? The answer is almost certainly *not* 5 %. Suppose the prevalence of exposure in the general population is 2 %, and suppose that if you have the condition then the probability that your blood test comes back positive is 90 % (the “power” of the test). Suppose we now test 1000 people. We would expect about 20 people to have actually been exposed and 18 to be correctly diagnosed. A total of 980 people have not been exposed but, due to random variation 5 %, i.e. 49, will be misdiagnosed. This means that a total of 18 + 49 = 67 will be diagnosed as having been exposed, of which only 18 really have, i.e. your chances of actually having been exposed is actually (18/67) or about one in four, not one in twenty. The reason for the difference in expectation and reality can be traced to a very basic misunderstanding: the “*Fallacy of the Transposed Conditional*” (*FTC*).

Suppose a person is described to you as being quiet and very organized and then you are asked whether this person is more likely to be a farmer or a librarian. Most people would chose the latter, and incorrectly. There are far more farmers in the world than librarians and so even though an average librarian is more likely be quiet and organized than an average farmer, the odds favor the person described being a farmer. This is an example of transposing a conditional. Suppose we agree to these general characterizations; in formal terms we would say:

$$p(quiet\;\& \;organized|librarian) > p(quiet\;\& \;organized|farmer)$$

Here, the “|” symbol should be read as the word “given”, e.g. the term on the left reads: the probability that a person is quiet and organized *given* they are a librarian. The *FTC* assumes that if the above statement is true then:

$$p(librarian|quiet\;\& \;organized) > p(farmer|quiet\;\& \;organized)$$

I.e. we assume that the probability someone is a librarian *given* they are quiet and organized is greater than the probability someone is a farmer *given* they are quiet and organized.To see why the *FTC* is to blame for misinterpreting the viral exposure test above, and much more besides, consider what we initially know:

$$p\left( {Test\;Positive|Not\;Exposed} \right) = 0.05$$

The *FTC* can lead us to think, therefore, that:

$$p\left( {Not\;Exposed|Test\;Positive} \right) = 0.05$$

Then, since someone can only have been exposed or not exposed:

$$p\left( {Exposed |Test\;Positive} \right) = 0.95$$

which is the naïve interpretation of being tested as positive.

The solution to the avoidance of the *FTC* was proposed by Bayes, and presented more formally by Laplace many years later. It is known as Bayes’ Equation and is, surely, one of the most profound equations [29].

82 $$p\left( {A|B} \right) = \frac{{p\left( {B|A} \right)p\left( A \right)}}{p\left( B \right)}$$

This merely says that to transpose the conditional we have to also multiply by the ratio of independent probabilities, or prevalences. In our examples of farmers and librarians this would look like:

$$p\left( {librarian|quiet\;\& \;organized} \right) = p(quiet\;\& \;organized|librarian)\frac{{p\left( {librarian} \right)}}{{p\left( {quiet\;\& \;organized} \right)}}$$

$$p\left( {farmer|quiet\;\& \;organized} \right) = p(quiet\;\& \;organized|farmer)\frac{{p\left( {farmer} \right)}}{{p\left( {quiet\;\& \;organized} \right)}}$$

As *p*(*farmer*) *≫* *p*(*librarian*) it is unlikely that, given the *data* of “*quiet&organized”* any intrinsic likelihood towards librarian (the first term on the right) is sufficient to win the argument. Similarly, for the viral exposure test:

$$p\left( {Exposed|Test\;Positive} \right) = \frac{{p\left( {Test\;Positive|Exposed} \right) \times p\left( {Exposed} \right)}}{{p\left( {Test\;Positive} \right)}}$$

83 $$\begin{aligned}& p\left( {Exposed|Test\;Positive} \right) \hfill \\& \quad= \frac{{p\left( {Test\;Positive|Exposed} \right) \times p\left( {Exposed} \right)}}{{p\left( {Test\;Positive|Exposed} \right) \times p\left( {Exposed} \right) + p\left( {Test\;Positive|Not\;Exposed} \right) \times p\left( {Exposed} \right)}} \hfill \\ &\quad= \frac{0.9 \times 0.02}{0.05 \times 0.98 + 0.9 \times 0.02} = \frac{0.018}{0.049 + 0.18} = \frac{18}{67} \hfill \\ \end{aligned}$$

Note that in this last line we have substituted all the essential quantities about our test, namely the significance (0.05), the power (0.9) and the prevalence (0.02), to arrive at the correct probability.

Does this have relevance to this article? At first glance it would seem that this is unrelated to calculating significance values or confidence regions. In fact, it is quite central. Much of what we take for granted in calculating error bars is, in fact, based on a *FTC*! In Part One we stressed how much of the foundation for statistical testing is based on the *Central Limit Theorem* (CLT). This states that an average will be distributed as a Gaussian, centered on the true average. Put formally, using the language of conditional probabilities, this looks like:

$$p(Observed|True\;Average) \propto Gaussian\left( {True\;Average} \right)$$

However, what we typically say when we calculate confidence limits using any of the techniques presented in this paper or its predecessor is:

$$p(True\;Average|Observed) \propto Gaussian\left( {Observed} \right)$$

This is *not* what the CLT says! Note that formal significance testing does not fall into this fallacy. Instead, it states that:

$$p(Observed|Null\;Hypothesis) \propto Gaussian\left( {Null\;Hypothesis} \right)$$

I.e., *given* the null hypothesis, the observed results will be distributed as a Gaussian around the average of that hypothesis. This is why statements describing significance testing often seem tortuous, e.g. the probability that an effect equal to or greater than that observed not being what we observed! As we have seen above, this does not save significance testing being misinterpreted; however strictly as defined it is formally correct. Not so for assuming that confidence limits naively follow from the CLT. And yet this is how error bars are typically presented, e.g. calculate an average and standard deviation from the observed data; can this be so wrong?

In fact, it is often *not* wrong. Just because transposing the conditional is formally incorrect does not mean doing so is always inappropiate. It depends, as in Bayes Theorem, on the unconditional probabilities, i.e. the probabilities as known before the data was observed.

$$p\left( {A|B} \right) = \frac{{p\left( {B|A} \right)p\left( A \right)}}{p\left( B \right)} = p\left( {B|A} \right) \quad if \quad p\left( A \right) = P\left( B \right)$$

Consider the case where we are comparing two methods, *A* and *B*. If we did not run any tests on the methods, i.e. we have no data with which to compare them, then we have no a priori reason to expect one to be better than the other, i.e. *p*(*A* > *B*) = *p*(*B* > *A*). Under these conditions, transposing the conditional is perfectly legal.

Even here, though, we have to be careful. Formally, Bayes tells us:

84 $$p\left( {Diff|data} \right) = \frac{{p\left( {data|Diff} \right) \times p\left( {Diff} \right)}}{{p\left( {data} \right)}} = \frac{{p\left( {data|Diff} \right) \times p\left( {Diff} \right)}}{{\smallint p\left( {data|Diff} \right) \times p\left( {Diff} \right)}}$$

where “Diff” is the difference in performance between two methods and “data” is the difference we observe. The LHS is the distribution of the actual difference *given* the data, i.e. the distribution on which we would base a confidence interval. Suppose we have the usual Gaussian distribution:

85 $$\begin{aligned}& p\left( {data|Diff} \right) = \left( {\sqrt {\frac{N}{{2\pi \sigma^{2} }}} e^{{ - \frac{{N\left( {\left\langle {data} \right\rangle - Diff} \right)^{2} }}{{2\theta_{data}^{2} }}}} } \right) \hfill \\& p\left( {Diff} \right) = constant = \delta \hfill \\ \end{aligned}$$

I.e. the first term in the numerator of Bayes’ Equation is as given by the CLT, the second just says that the a priori probability of seeing a given difference is a constant proportional to the (small) width, *δ*, we associate with this difference. Then the integral on the denominator is simply the normalization of the CLT Gaussian, i.e. equal to one. Therefore:

86 $$p(Diff|data) = \left( {\sqrt {\frac{N}{{2\pi \sigma^{2} }}} e^{{ - \frac{{N\left( {Diff - \left\langle {data} \right\rangle } \right)^{2} }}{{2\theta_{data}^{2} }}}} } \right)\delta$$

Here the ordering of “data” and “Diff” in the exponential has been switched just to give emphasis to this being a formula for the distribution of “Diff”, i.e. the difference between the observed and actual difference. This is exactly what we are typically *incorrectly* taught the CLT proposes for error bars/confidence limits on a measurement. It is incorrect because consider if *p*(*Diff*) is not a constant. After all, do we really expect any difference in methods to be equally likely? We probably expect a small difference to be more likely. This is information that is ignored in the *FTC* and can actually affect the error bars.

More importantly is the case in which *p*(*Diff*) is non-symmetric, i.e. we have reason to believe that *A* is superior to *B* even before we take any measurements. Although outside the scope of this article, adding prior information in favor of one method simply makes it more difficult for the less favored method to ‘prove’ itself. For example, many docking programs have been around for a long time, do we really expect some new method to give radically better results? Perhaps, but it would seem reasonable to evince some skepticism unless presented with over-whelming evidence. Bayes provides a way to incorporate both this evidence and that skepticism. In general, such prior information acts as a ‘deflator’ of the observed difference. Although this might seem to only apply to the comparison of computational methods it also applies to experimental measurements. For instance, if we have empirical evidence that it is less likely to find highly active compounds than less active compounds it is necessary to adjust observed affinities lower by Bayes reasoning.

Recently the Bayesian community has provided a powerful framework for reevaluating *pvalues* in terms of odds-ratios [30]. An odds-ratio is just the ratio of the odds in favor of observations being derived from one mechanism as opposed to the odds in favor of a distinct and different mechanism. This is a quite radical revision because it puts the classical “null hypothesis” on the same footing as the competing hypothesis and merely asks, “Which is more likely?” Non-experts might well say, “Well, isn’t this exactly what significance testing does?” It is certainly how it is often presented; however, actually what is being provided are the odds against the null hypothesis, not *for* the new hypothesis. Odds-ratios correct this balance and their lack is being proposed as one of the major reasons that many papers published quoting *pvalues* for a particular effect are often found to be wrong in subsequent publications. As a motivating example, this approach would suggest that a finding that is significant at the *p* = 0.05 level is actually likely to be incorrect about one-third of the time!

A précis of this revision is as follows: Suppose some effect is seen after some treatment. We have experience of a null model, e.g. placebo or no treatment, and so know the likelihood this could produce this observation—this is to say, the traditional *pvalue*. However, what is the probability a new mechanism is producing this effect? As above, we need to have a priori estimates for both the likelihood that something different is happening, e.g. actual viral exposure in the viral exposure test considered earlier, and the likelihood that this difference can produce the effect. For simplicity let us assume that it is equally likely that a new mechanism is in play as is not, e.g. perhaps we’ve prescribed a medicine that we think will affect a particular pathway that will produce an etiology, but we are not 100 % certain, just 50 % sure. The second question is *if* that pathway is being affected, could it produce the observed effect? Of course, we don’t really know this, in other words, we don’t know what the likely size of effect may be (that is, after all, why we do the experiment).

Let us consider two extremes. The first is that the probability for the size of effect of the new mechanism is sharply peaked around zero, i.e. there is little probability of producing the effect seen. For this example the odds-ratio would favor the null-model, which at least has some probability of randomly producing the observed outcome. Now consider a second model in which we consider it equally likely for the new mechanism to produce a small, medium, large or even extra-large effect. Ironically, this may *also* lead to an odds-ratio in favor of the null-model, simply because the probability distribution for the new mechanism does not favor any particular value—it is just as likely to be predicting a larger effect as a smaller one. This suggests that there must be some “golden mean”, e.g. some distribution between one sharply peaked around zero and one with little discrimination that favors the new hypothesis. Remarkably, this can be formally defined, i.e. we can find the best probability function for the new effect, given no prior information. This ‘best’ function produces the best possible odds-ratio in favor of the new versus the old mechanism after seeing the evidence. Even more remarkably there is a simple formula by which to convert from the traditional *pvalue* to the new odds-ratio in favor of the new mechanism:

87 $$O = odds - ratio = - \frac{1}{e \times p \times \ln \left( p \right)}$$

For instance, if *p* = 0.05, then *O* = 2.45, i.e. the odds in favor of the new mechanism are roughly two and half times as high as those for the null model. Given that the probabilities for either mechanism producing the effect must sum to one, this means the probability of the effect being produced by the null model, rather than the new mechanism is 1.0/(2.45 + 1) or about 29 %, i.e. much higher than the expected 5 %.

Suppose we actually do have an estimate as to whether a new pathway is being affected, and this probability is *q*. Then the Bayesian odds-ratio is modified in a very simple way, namely:

88 $$O\left( q \right) = odds - ratio = - \frac{q}{e \times p \times \ln \left( p \right)}$$

I.e. the odds-ratio diminshes as a linear function of the prior probability of there being a new effect.

Does this matter in the comparison of different methods or approaches? Largely it does not. This application of Bayes research applies when we do not know what is responsible for an observation, mechanism one or mechanism two. The typical use of statistics in this paper is not aimed at whether an effect exists or not but whether we can expect one method to be better or not. None-the-less, it is important to be aware as to the general limitations of the tools we use, whether they are *pvalues* or error bars.

**Fig. 9:**
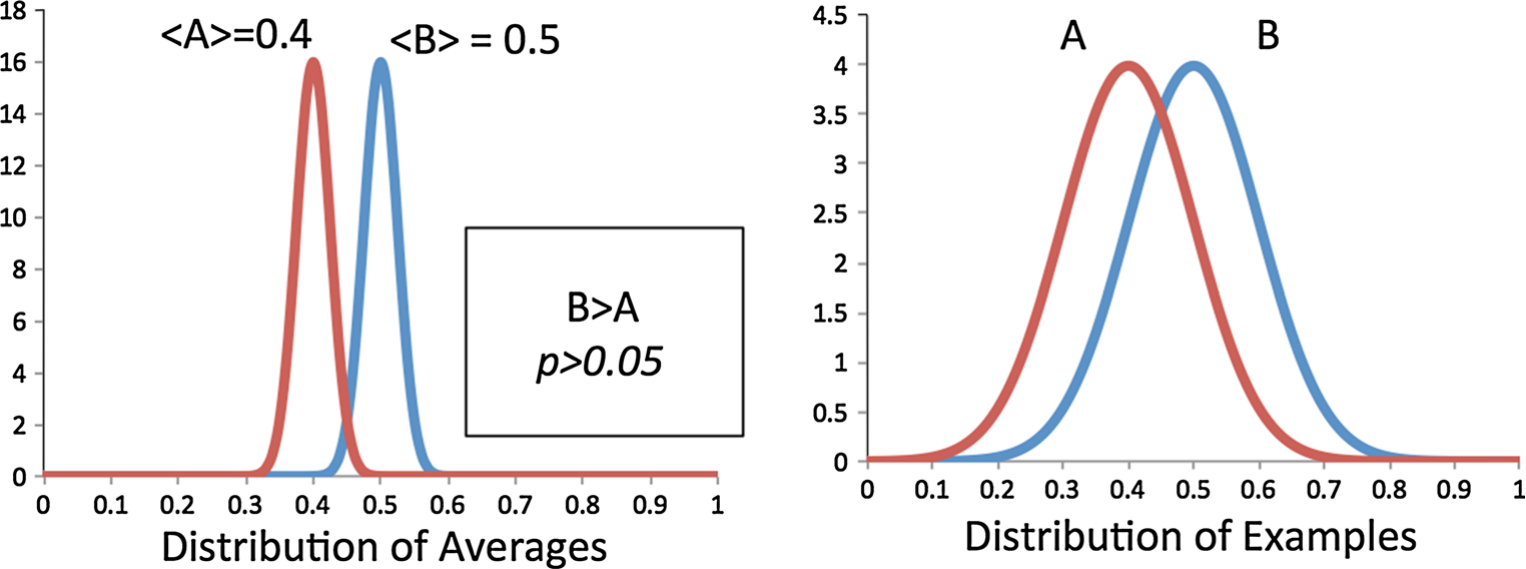
Illustration of the difference between the averages of two properties compared to their distributions. The distribution of averages changes with the number of observations (*sharpens* as that number increases), whereas the distributions are intrinsic properties that become stable with respect to increasing sample size

A final concern regarding significance testing has nothing to do with any application of Bayes but is perhaps the most important, and that is the issue of size-of-effect [31]. In Fig. 9 we illustrate this distinction. If the number of observations on *A* and *B* increases then the averages become better defined and the significance of the difference in properties becomes greater (left panel). However, on the right is shown the distribution of effects and these distributions. Other than becoming better defined, these do not change with sample size. What are the essential features of *A* versus *B*? Is it the certainty with which we can say that *B* is larger than *A*? Or is it the difference between the averages? Or is it the overlap of the distributions illustrated in the right-hand panel?

In an attempt to quantify the difference between *A* and *B* that does not depend on sample size, i.e. is intrinsic to both, Cohen defined a metric, *d* that bears his name [32]:

89 $$d = \frac{{\left| {\left\langle B \right\rangle - \left\langle A \right\rangle } \right|}}{{\sqrt {\sigma_{A}^{2} + \sigma_{B}^{2} } }}$$

Note that this looks similar to a *t* statistics for Student’s *t* test except it lacks any sample size, *N*. Cohen suggested that *d* form a rough guide to the importance of the size of effect, e.g. whether the improvement going from *A* to *B* is significant (in a real world use of the word ‘significant’). As applied in the social sciences, he recommended treating effects with a *d* less than 0.2 as unimportant, those between 0.2 and 0.5 as of minor importance, between 0.5 and 0.8 as significant and greater and 0.8 as important. In Part One we considered ways to calculate the error bars on such a quantity.

Cohen’s *d* is important because it does capture aspects missing from significance testing on the average properties. However, it is also a little unclear as to why Cohen’s ranges are given their designations. In this author’s opinion it is more useful to relate *d* to the probability that one method will outperform the other on the *next* application. That is to say, if the performance of *A* on the next system is a random draw from its distribution and that of *B* from its distribution, what is the probability that *B* outperforms *A*? This is clearly related to both the widths of the distributions and the separation of the peaks. This probability can be expressed in terms of the *erf* function. However, if the widths of the respective distributions are roughly comparable then we can reinterpret *d* in the range from zero to one as:

90 $$p\left( {B > A} \right) \approx 0.25d + 0.5$$

So, if *d* = 0.2 we have *p*(*B* > *A*) = 0.55 or *p*(*A* > *B*) = 0.45, i.e. *B* will outperform *A* eleven times out of twenty. If *d* = 0.5 we have *p*(*B* > *A*) = 0.625 or *p*(*A* > *B*) = 0.375, i.e. *B* will outperform *A* five times out of eight. If *d* = 0.8 we have *p*(*B* > *A*) = 0.7 or *p*(*A* > *B*) = 0.3, i.e. *B* will outperform *A* seven times out of ten. Not only does this give a more concrete feel to what Cohen’s *d* actually represents, these are potentially useful numbers. In the case of *d* = 0.8 we know that the odds in favor of *B* are more than 2:1 and unless other characteristics are more important (e.g. expense or time) *B* is the method of choice. However, if *d* = 0.2 the advantage is slim. If there are compensating advantages to method *A* it may well be the better choice.

It seems appropriate to end this section with this consideration, i.e. whether a method is better than another, or even how much better, may not be as important as the probability it gives superior performance on the *next* application. This is an aspect of a pair of methods that can be applied to any utility function involving cost, speed, expertise, and resources and so forth such that a judicious choice can be made of *utility*. Exercises in assessing utility are rare, although not without precedence [33]. There are more ways to progress in computational chemistry than just small increments in performance, a broader context is important. All the examples in the previous sections that attempt to provide error bars on methods are, therefore, important also in providing distributions of properties or differences and should be considered necessary tools for the practicing computational chemist trying to make an informed decision.

## Conclusions

The field of computational chemistry is an empirical field; statistical issues abound, from the quality of training sets to the high variability of the performance of methods from case study to case study, to the consideration of true utility in the context of drug discovery and design. This paper, and the one before it, has tried to present a clear framework for at least calculating error bars on computational or empirical quantities. With error bars one at least has a sense of whether knowledge is precise or vague, or even the precision with which we know how vague something really is. These would seem essential to a field to judge when new methods should supersede old ones, and to provide nuance to predictions made with either.

Although there are issues to be considered in the evaluation of either *pvalues* or confidence limits, they both are useful concepts, even if they need to be considered with care. In particular, as comparisons are increasingly made on common datasets, approaches to handle correlations between methods are important. Clear winners will still be clear winners, but more subtle improvements, as often occur as methods are continuously refined, need more careful consideration. This paper briefly considered some of the effects of parameterization on performance, e.g. more parameters will reduce training set error, but at a potential cost to future performance, and analytic approximations to this risk. In fact, multiple hypotheses testing as described herein is an implicit form of parameterization that is often not recognized. There is insufficient space in this article to begin to address the many techniques from both information theory and Bayesian theory that exist to address over-parameterization.

Finally, careful consideration of prior knowledge and the incorporation of such into a testing framework, and the appreciation for the importance of intrinsic variability, as in the size-of-effect parameter from Cohen, are important aspects for our field to consider. Both require careful thought, yet both are richer ways of looking at data and the world around us.
